# Linkages between *Sphagnum* metabolites and peatland CO_2_ uptake are sensitive to seasonality in warming trends

**DOI:** 10.1111/nph.18601

**Published:** 2022-12-07

**Authors:** Anna Sytiuk, Samuel Hamard, Régis Céréghino, Ellen Dorrepaal, Honorine Geissel, Martin Küttim, Mariusz Lamentowicz, Eeva Stiina Tuittila, Vincent E. J. Jassey

**Affiliations:** ^1^ Laboratoire Ecologie Fonctionnelle et Environnement (LEFE) Université Paul Sabatier, CNRS F‐31000 Toulouse France; ^2^ Department of Ecology and Environmental Science, Climate Impacts Research Centre Umeå University SE‐981 07 Abisko Sweden; ^3^ Institute of Ecology, School of Natural Sciences and Health Tallinn University Uus‐Sadama 5 10120 Tallinn Estonia; ^4^ Climate Change Ecology Research Unit, Faculty of Geographical and Geological Sciences Adam Mickiewicz University in Poznań Bogumiła Krygowskiego 10 61‐680 Poznań Poland; ^5^ School of Forest Sciences University of Eastern Finland Joensuu Campus FI‐80100 Joensuu Finland

**Keywords:** carbon cycle, climate change, climate feedback, intraspecific variability, phenotypic plasticity, plant metabolism, seasonality, *Sphagnum*

## Abstract

- Plants produce a wide diversity of metabolites. Yet, our understanding of how shifts in plant metabolites as a response to climate change feedback on ecosystem processes remains scarce. Here, we test to what extent climate warming shifts the seasonality of metabolites produced by *Sphagnum* mosses, and what are the consequences of these shifts for peatland C uptake.
- We used a reciprocal transplant experiment along a climate gradient in Europe to simulate climate change. We evaluated the responses of primary and secondary metabolites in five *Sphagnum* species and related their responses to gross ecosystem productivity (GEP).
- When transplanted to a warmer climate, *Sphagnum* species showed consistent responses to warming, with an upregulation of either their primary or secondary metabolite according to seasons. Moreover, these shifts were correlated to changes in GEP, especially in spring and autumn.
- Our results indicate that the *Sphagnum* metabolome is very plastic and sensitive to warming. We also show that warming‐induced changes in the seasonality of *Sphagnum* metabolites have consequences on peatland GEP. Our findings demonstrate the capacity for plant metabolic plasticity to impact ecosystem C processes and reveal a further mechanism through which *Sphagnum* could shape peatland responses to climate change.

## Introduction

Current and future climate change is anticipated to influence the carbon (C) dynamic of terrestrial systems (Frank *et al*., 2015), with the most pronounced effects expected at high northern latitudes (Ito *et al*., 2020). Among northern ecosystems, peatlands are particularly vulnerable to increasing temperatures and shifts in precipitation patterns because biological and chemical processes are more temperature‐ and water‐sensitive in these environments (Charman *et al*., 2013; Loisel & Yu, 2013; Weedon *et al*., 2013; Jassey & Signarbieux, 2019). Peatlands represent a major global C stock that is sensitive to climate change (Dorrepaal *et al*., 2009; Heijmans *et al*., 2013; Wilson *et al*., 2016; Hopple *et al*., 2020). Warming, along with corresponding changes in hydrology, may stimulate the return of the stored belowground C to the atmosphere as CO_2_ (Dorrepaal *et al*., 2009; Bragazza *et al*., 2016) and/or methane (Bridgham *et al*., 2013; Hopple *et al*., 2020), further amplifying climate change. However, these losses of C to the atmosphere often do not consider the potential changes in the timing of seasonal events, which are likely to increase plant productivity (Hajek & Knapp, 2022). As climate change particularly alters the seasonal patterns of temperature and precipitation in northern latitudes (Fischer & Knutti, 2016; Wang *et al*., 2017; Santer *et al*., 2018), plant phenology and physiology may be affected by climate change. At a fundamental level, earlier spring green‐up and delayed onset of autumn dormancy resulting from the rising temperature at northern latitudes (Park *et al*., 2016) may increase vegetation C uptake over the growing season, thus potentially balancing the net effect of climate change on peatland C dynamic (Loisel *et al*., 2012; Gallego‐Sala *et al*., 2018). Therefore, the ways in which the vegetation responds to the changes in the seasonal timing and synchrony of climatic events – or seasonality – are central to understanding and predicting peatland responses to global change.

Plants underpin the peatland C cycle both as important C fixers and as the main source of dead organic matter that fuels microorganisms (van Breemen, 1995; Turetsky, 2003a; Rydin & Jeglum, 2013). In particular, *Sphagnum* mosses effectively facilitate wet, anoxic and acidic conditions that inhibit decomposition and thus favour C sequestration (Van Breemen, 1995; Turetsky, 2003b). Hence, predicting the response of *Sphagnum* mosses to climatic change is essential for the assessment of the response of the peatland C cycling. Over the past decades, climate change has been shown to influence *Sphagnum* mosses, with a possible decrease in their cover at the expense of vascular plants (Jassey *et al*., 2013, 2018; Buttler *et al*., 2015; Dieleman *et al*., 2015; Lyons *et al*., 2020), potentially resulting in higher respiration (Ward *et al*., 2013; Kuiper *et al*., 2014; Jassey *et al*., 2018) and litter decomposition rates (Ward *et al*., 2015; Ofiti *et al*., 2022). Additionally, it is well established that *Sphagnum* productivity declines with increasing temperature (Bragazza *et al*., 2016; Norby *et al*., 2019), although such decline is strongly related to seasonality in water availability (Jassey & Signarbieux, 2019). These last findings suggested that *Sphagnum* mosses can adjust to changing climate through phenotypic plasticity and may adapt to climate change in future (Gallego‐Sala *et al*., 2018). Therefore, gaining an understanding of the mechanisms of *Sphagnum* phenotypic plasticity is crucial for predicting changes in peatland species distributions, plant community composition and primary productivity under climate change.

Phenotypic plasticity in a given plant species is often mediated at the molecular level at first (Peñuelas *et al*., 2013; Gargallo‐Garriga *et al*., 2015; Peters *et al*., 2018). Plants produce a tremendous diversity of metabolites (Fernie *et al*., 2004), which play diverse roles in plant growth and survival (Berini *et al*., 2018; Peters *et al*., 2018). Because plant metabolites lie at the interface between genes and the environment (Peters *et al*., 2018; Walker *et al*., 2022), they are unique for encompassing the physiological processes maximizing plant fitness across timescales, from hours to seasons to generations (Peñuelas *et al*., 2013). Therefore, plant metabolites are key for detecting the phenotypic response of the organism under climate change, and the metabolic pathways that are up‐ and downregulated in response to climate changes (Gargallo‐Garriga *et al*., 2015). For instance, exposure to drought and/or warming can lead to the accumulation of primary metabolites, including proline, sugars, amino acids and proteins associated with photosynthesis, as well as secondary metabolites such as phenolics, flavonoids and tannins (see references in Peñuelas *et al*., 2013 and Laoué *et al*., 2022). Climate change can therefore have a positive effect on plant growth and primary production, but can also negatively affect plant performance as a result of metabolic plasticity. In peatlands, the effects of warming and/or drought on *Sphagnum* metabolites showed contrasting effects on primary and secondary metabolites (Dorrepaal *et al*., 2005; Jassey *et al*., 2011a, 2013; Reczuga *et al*., 2018; Rastogi *et al*., 2020; Antala *et al*., 2022), suggesting that taxonomy and seasonality are important forces determining metabolic plasticity. Hence, investigating the effects of climate change on *Sphagnum* metabolites across species and over seasons is needed, as well as understanding the relative importance of *Sphagnum* metabolic plasticity in dictating the response of peatland C uptake to climate change.

Our aims were to explore whether climate change causes intraspecific variation in *Sphagnum* metabolites across seasons, and how this ultimately influences peatland C uptake. We established a reciprocal transplant experiment on a latitudinal gradient across five European *Sphagnum*‐dominated peatlands to test the main hypothesis that (1) exposure to climate change induces metabolic plasticity in *Sphagnum* mosses, resulting in effects on peatland CO_2_ uptake. We also test the hypothesis that (2) the effect of climate change on *Sphagnum* metabolic plasticity depends on seasonality and so the effect on peatland C uptake. Particularly, we expect that climate change will initiate trade‐offs of resource partitioning between metabolites involved in the growth (i.e. primary metabolites) or the survival (i.e. secondary metabolites) of *Sphagnum* mosses across seasons. Finally, we test the hypothesis that (3) *Sphagnum* metabolic plasticity depends on taxonomy, with different effects on peatland C uptake according to species. We specifically monitored shifts in *Sphagnum* metabolites by quantifying broad classes of primary and secondary metabolites, alongside local rates of gross photosynthesis. Reciprocal transplant experiments have been extensively used to test for the direct effect of temperature on plants (Breeuwer *et al*., 2010; Alexander *et al*., 2015; Walker *et al*., 2019). Here, by linking a reciprocal transplant experiment to a metabolic approach on multiple sites and species, we were able to not only quantify temperature and precipitation change effects on *Sphagnum* phenotypes and the C cycling but also overcame the difficulty of disentangling biotic and abiotic effects on peatland C uptake.

## Materials and Methods

### Experimental set‐up and sampling

**Fig. 1:**
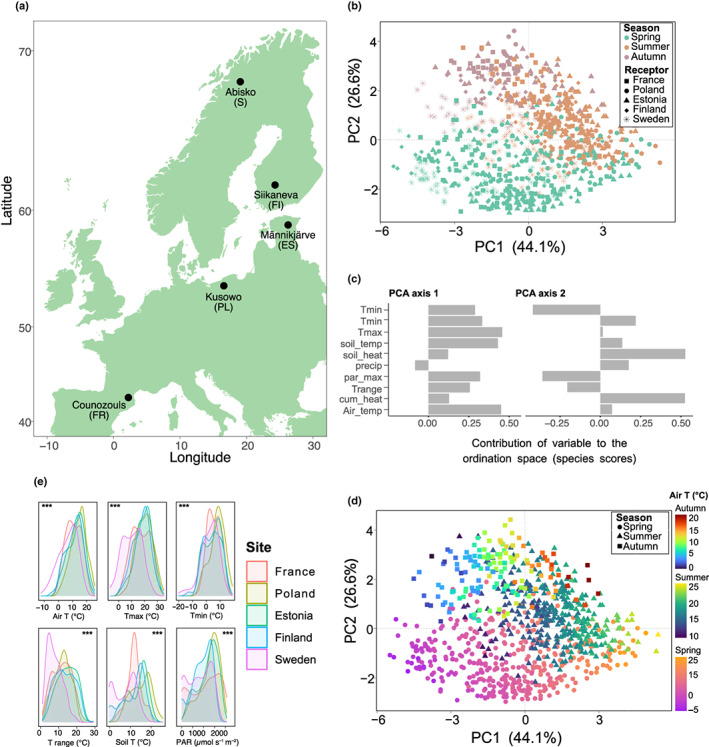
Locations and characteristics of the transplant gradient. (a) The transplant experiment was established in five European *Sphagnum*‐dominated peatlands. Capital letters indicate the country of origin: ES, Estonia; FI, Finland; FR, France; PL, Poland; S, Sweden. (b) Principal component analysis (PCA) of climatic data collected in each site with receptor sites represented as shapes and seasons as colours. (c) The loadings of each climatic factor on the two first PCA axes: Tmin, minimum air daily temperature; Tmax, maximum air daily temperature; soil_temp, mean daily soil temperature; soil_heat, cumulative soil temperature; precip, daily precipitation; par_max, maximum daily PAR; Trange, diurnal temperature range (maximum–minimum daily temperature); cum_heat, cumulative air temperature; Air_temp, mean air daily temperature. (d) Principal component analysis of climatic data collected in each site with seasons represented as shapes and air temperature as colour gradients. (e) Kernel density estimates showing the distributions of selected climatic variables for each site. Asterisks illustrate significant differences (***, *P* < 0.001; linear models, ANOVA) between sites.

We conducted a reciprocal transplant experiment across five European peatlands distributed along a latitudinal gradient, from northern Sweden to southern France (Fig. 1a). We selected five *Sphagnum*‐dominated peatland sites – that is Sweden, Finland, Estonia, Poland and France – to represent a wide temperature (temperature gradient = 10°C) and precipitation (precipitation gradient = 200 mm) range within Europe (Supporting Information Table S1). Each site was dominated by different *Sphagnum* and vascular plant species (see Sytiuk *et al*., 2021, 2022 for a detailed description of each site). In the summer of 2018, we collected 25 peat‐monoliths (60 × 40 × 20 cm) at each site and encased them into plastic boxes of the same size. Plastic boxes were sterilized beforehand, and we made holes sealed with 40 μm pore nylon mesh in their bottom to allow for the transport of water and nutrient while preventing the immigration of microbial cells from the bare peat as much as possible. The transplantation of all peat‐mesocosms was completed within 18 d under cool conditions for the boxes. The water table depth of each mesocosm was maintained at its field level during transport using water from their respective site. In each site, five homogeneous blocks were defined; in each block, five peat‐monoliths with homogeneous *Sphagnum* carpets have been selected. Five peat‐mesocosms (one from each block) stayed at their original location but in the plastic boxes, while other peat‐mesocosms were dispatched among the four other sites and thus experienced warmer/colder and/or wetter/drier climate, depending on the origin site (in total: 5 sites × 5 blocks × 5 replicates = 125 peat‐mesocosms). In addition to the transplanted plots, we selected five untouched plots, one in each block, as control of the box effect in each site (in total: 5 sites × 5 replicates = 25 untouched plots). Following transplantation, we monitored *Sphagnum* growth in peat‐mesocosms using the cranked wire method (Küttim *et al*., 2020) to assess *Sphagnum* acclimation. All *Sphagnum* species grew in peat‐mesocosm (Fig. S1), showing a good acclimation to new environmental conditions.

In spring 2019, we started seasonal monitoring of *Sphagnum* metabolites and gross ecosystem productivity (GEP) in each peat‐mesocosm and untouched plot, in each site. Three sampling campaigns were performed in spring (from 16 April to 22 May 2019), summer (from 3 to 27 July 2019) and autumn 2019 (from 30 September to 25 October 2019) across all sites. In each peat‐mesocosm and untouched plot, we collected the dominant *Sphagnum* species at the site of origin for measuring metabolites contents: *S. warnstorfii* R. for France mesocosms, *S. magellanicum* B. for Poland mesocosms, *S. rubellum* W. for Estonia mesocosms, *S. papillosum* L. for Finland mesocosms and *S. balticum* R. for Sweden mesocosms, respectively. For each campaign and in each peat‐mesocosm and untouched plot, *Sphagnum* shoots were sampled around three permanently marked spots (*c*. 3–5 shoots per spot). This sampling design allowed us to obtain a composite sample, which represented the entire mesocosm (or untouched plot). Immediately after sampling, the top of the *Sphagnum* shoots (0–3 cm) was cut, pooled, dispatched for the different laboratory analyses and stored at 4°C until we came back to the laboratory. No samples have been collected from the Swedish peat‐mesocosms in autumn because they were frozen and sampling would have damaged our plots.

### Collection of environmental data and vegetation cover

Daily air and soil temperature, precipitation and photosynthetically active radiation (PAR) at the *Sphagnum* carpet level were measured at hourly intervals at each site (Meter^®^ sensors and data loggers; Meter Group, Pullman, WA, USA). In addition to microclimatic data, we monitored carbon and nitrogen availability on *Sphagnum* carpet using the water‐extractable organic matter (WEOM) approach. Briefly, and according to Jassey *et al*. (2018), *Sphagnum* shoots (*c*. 3 g of fresh weight, 0–3 cm to the capitula) were soaked in 30 ml of demineralized water followed by 90 min of shaking at 150 rpm. Then, *Sphagnum* shoots were dried at 60°C for 48 h and weighed to obtain dry mass (mg g^−1^ DW). The water extract was filtered (Whatman™ filters, 1 μm pore size; Cytivia, Marlborough, MA, USA), and dissolved organic carbon and nitrogen (WEOC and WEON, respectively) were quantified using a TOC analyser. Moreover, we took water samples in each peat‐mesocosm and untouched plot to measure pH in boxes and test whether transplantation influenced the pH of the peat‐monoliths. We did these measurements only in three receptor sites (Finland, Estonia and France). We did not find any effect of transplantation on pH in peat‐mesocosms, showing that the pH in peat‐mesocosms transplanted in other sites remained similar to its donor site (Fig. S1; Table S2), thus excluding pH as a potential driver of shifts in *Sphagnum* metabolites. Further, we measured the water content (WC) of every *Sphagnum* species in each season by collecting *c*. 3 g of fresh moss on the day of sampling, weighing it fresh and drying it for 2 d at 60°C. The *Sphagnum* WC was expressed in grams of H_2_O per dry mass of bryophyte (g H_2_O g^−1^ dm). To estimate the relative cover of *Sphagnum* and vascular plants in each plot (mesocosms and untouched plots), we took two high‐resolution photographs for each half of the plot and analysed the pictures following Buttler *et al*. (2015).

### *Sphagnum* metabolite analyses

We quantified a set of nine *Sphagnum* primary and secondary metabolites according to Sytiuk *et al*. (2022): Chl*a* and Chl*b*, carotenoids, total carbohydrates, total phenols, total flavonoids, proline, water‐soluble phenolics and total tannins. Briefly, *Sphagnum* mosses were frozen, lyophilized, ground and stored at −20°C before chemical analyses. Then, we used different extractions to quantify these different compounds: (1) a 99.9% methanol extraction for quantifying *Sphagnum* pigments (Chl*a*, Chl*b* and total carotenoids), (2) a 50% methanol extraction for quantifying total phenols, flavonoids, tannins and carbohydrates, (3) a water extraction for quantifying water‐soluble phenolics and (4) a sulfosalicylic acid extraction for quantifying proline. All metabolites were quantified with spectroscopy using different wavelengths (Chl*a*, Chl*b* and carotenoids at 480, 652 and 665 nm; total carbohydrates at 490 nm; total phenols at 760 nm; total flavonoids at 595 nm; total tannins at 500 nm; and total proline at 510 nm) and standards. All methodological details are given in Sytiuk *et al*. (2022).

### Gross ecosystem productivity measurements

Gross ecosystem productivity was measured in each peat‐mesocosm and untouched plot during each field campaign under optimal sunlight (sunny weather, between 10:00 h and 13:00 h) using a portable infrared gas analyser (Targas‐1; PP‐System, Amesbury, MA, USA) equipped with a CPY‐5 transparent canopy chamber. CO_2_ measurements have been performed under an airtight seal within the chamber through the use of a custom‐made PVC collar installed in each plot. The record of CO_2_ concentrations was set every second for 90 s in order to avoid the build‐up of heat and condensation inside the chamber. Net ecosystem CO_2_ exchanges (NEE) were measured with the transparent CPY‐5 chamber, whereas ecosystem respiration (ER) was assessed using a darken CPY‐5 chamber. All fluxes were measured at ambient temperature and light conditions at each site. We calculated CO_2_ fluxes as a linear change in CO_2_ concentration (ppm) over the measurement period using the R package gasfluxes (Fuss *et al*., 2020). According to the gasfluxes, we took into account ambient atmospheric pressure, soil temperature, the volume of the CPY‐5 chamber, the surface area of the PVC collar and light intensity. For each field campaign and in each plot, we quantified CO_2_ fluxes for 2–3 consecutive days, calculated the flux rates and used the average. Gross ecosystem productivity – CO_2_ uptake as a result of photosynthesis – was calculated as the difference between NEE and ER. Negative GEP values indicate CO_2_ uptake, while positive values CO_2_ release. Because vascular plant cover could vary among blocks and across sites, we standardized GEP data by the relative vascular plant cover (per cent cover estimates) to obtain comparable fluxes among all mesocosms.

### Statistical analyses

All data and statistical analyses were performed in R v.3.5.3 (R Core Team, 2019) using specific packages, as indicated later.

We performed a principal component analysis (PCA) to assess the global and seasonal climatic patterns of each site. From our daily measurements of temperature and precipitation in each site, we calculated different metrics: mean, minimum and maximum daily air and soil temperature, daily precipitation, temperature diurnal range as the difference between the maximum and minimum air temperature, and cumulative air and soil heat between 1 March and 31 October. In addition to the PCA, we used kernel density plots of key climatic variables to visualise the distribution of data over a continuous time period in each site.

We tested the effect of transplantation over seasons on *Sphagnum* metabolites, GEP, *Sphagnum* WC and WEOM data using linear mixed‐effects models (LME; lme4 R package). The models were fitted with the *Sphagnum* species, receptor site and season as fixed effects and with the plot nested into block and receptor site as a random effect on the intercept to take into account potential pseudoreplication resulting from repeated measurements over time in the same plot and site (Pinheiro & Bates, 2000). Additionally, we ran another set of LME models to test the effects of mean temperature, precipitation, delta temperature (i.e. the difference in temperature between the reference site and other sites) and delta precipitation (i.e. the difference in precipitation between the reference site and other sites) on *Sphagnum* metabolites, GEP, *Sphagnum* WC and WEOM. The coldest site, that is the Swedish site, was used as a reference to calculate delta temperature (∆temperature) and precipitation (∆precipitation) among sites. These LME models used the same random factor as described above. Climatic variables were retrieved from our weather station at each site. For each season, we calculated the mean (or the sum for precipitation) of every climatic variable (e.g. air temperature) over a period of 30‐d preceding sampling (Table S1; Fig. S2). Finally, we used different sets of LME models to test the robustness of our experimental design. First, we tested for the ‘box’ effect on our different variables by fitting LME models with ‘box’ (i.e. with or without box), species and season as fixed effects, and plot nested into species and season as a random factor. The box effect in each site was not significant for most of the metabolites and *Sphagnum* species over the three seasons (see Fig. S3; Table S2 for details), as well as for CO_2_ fluxes (Fig. S4; Table S3). Second, we used LME models to assess the effect of transplantation on the pH in the transplanted peat‐mesocosms (fixed effect), with plot as a random factor of the intercept. pH values in transplanted peat‐mesocosms did not significantly change following transplantation and remained similar to their donor site (Fig. S5; Table S4). Tukey multiple comparison tests were used for *post hoc* analyses of differences among the levels of the fixed effects in every LME model. Normality and homogeneity assumptions of the data, as well as of model residuals, were assessed using a Shapiro test and diagnostic plots. Log_10_ transformations of the data were applied if needed in order to meet these assumptions.

We used PCA to assess the global seasonal and transplantation effects on *Sphagnum* metabolites composition; we used a standardized transformation with *decostand* function from vegan package beforehand. Site scores from the PCA were further extracted for the two first axes to correlate shifts in *Sphagnum* metabolites composition at each season with GEP. In addition, we used redundancy analyses (RDA) to assess how environmental parameters (temperature, precipitation, delta temperature and delta precipitation and WEOM) drove the distribution of *Sphagnum* metabolites across seasons and the transplant gradient. Adjusted *R* ^2^ was used to estimate the proportion of explained variance (Peres‐Neto *et al*., 2006). The significance of each explanatory variable included in RDA was tested using 1000 permutations. Variance partitioning (vegan package; Oksanen *et al*., 2019) was used to determine the relative importance of environmental variables on *Sphagnum* metabolites. We also used the variation inflation factor (*vif.cca*) to analyse linear dependencies among explanatory variables.

To analyse the metabolic plasticity of each *Sphagnum* species in response to reciprocal transplantation (i.e. reaction norms), we used random regression mixed‐effects models (RRMMs; lme4 package). Following the methodology described by Arnold *et al*. (2019), we tested seven models, including linear and quadric models with random factors varying in intercept, slope and/or curvature, with the site scores from axis 1 or 2 of the PCA performed on *Sphagnum* metabolites as response variables and cumulated temperature or precipitation as fixed variables. Random effects including receptor site and *Sphagnum* species were used. Maximum likelihood (REML = FALSE) was used to fit models and ensure that models with different fixed effects can be compared directly (Zuur *et al*., 2009). Additionally, we compared the fit of the models through *R* ^2^ values (Nakagawa & Schielzeth, 2013), Akaike information criterion (AIC) (Burnham & Anderson, 2004) comparing a log‐likelihoods of the models, and a likelihood ratio test. The outputs of tested models are shown in Tables S5 and S6. To visually assess how well the RRMM models fitted the raw data, we overlayed the regression line from the best model as an average population‐level reaction norm using the *predict* function. Finally, we used best linear unbiased predictors (BLUPs) to identify the phenotypic plasticity of *Sphagnum* species against temperature or precipitation variations (Arnold *et al*., 2019). Best linear unbiased predictors are based on the difference between the random regression slope coefficient of the RRMM for individual *Sphagnum* species and the predicted average of all five species. *Sphagnum* species have been ranked in order of plasticity according to BLUP slope estimates (i.e. the degree of plasticity).

## Results

### Climatic conditions and edaphic parameters

Climate conditions strongly varied among the five sites and across seasons (Fig. 1). The PCA evidenced that spring was relatively wet in most sites with strong air and soil temperature variations (Fig. 1a,b). Summer was relatively dry and warm in most sites, while autumn was the wettest season in most sites with relatively cold temperatures (Fig. 1b,c; Table S1). Our PCA further revealed net warming effects within each season across the latitudinal gradient (Fig. 1d). The five sites collectively captured a large temperature gradient across seasons and showed slight but significant differences between them (Fig. 1e; Table S1). In particular, air temperature ranged from 1.3°C to 8.1°C in spring, 11.1°C to 19.8°C in summer and 2.9°C to 8.9°C in autumn across the five sites (Table S1), thus representing a temperature variation across sites of 7.3°C on average and over seasons. Overall, Poland was the warmest site across seasons, both in terms of mean, maximum and minimum temperatures, while Sweden was the coldest site (Fig. 1c,d; Table S1). France, Estonia and Finland were relatively similar in terms of temperatures (Fig. 1e), although Estonia and Finland showed higher mean and maximum temperatures in spring than France (Table S1). However, France, together with Poland, showed more stable temperatures (lower diurnal ranges) than Estonia and Finland, resulting in lower variability in soil temperature, particularly in spring. In terms of precipitation, cumulated precipitation also varied among sites and between seasons. It ranged from 11 to 129 mm in spring, 29 and 64 mm in summer and 69 and 201 mm in autumn (Table S1). France was the wettest site and Finland the driest. In terms of light availability (PAR), we found differences among sites and between seasons (Fig. 1e; Table S1). However, overall PAR values were high enough in each site to ensure optimum *Sphagnum* growth across seasons.

The *Sphagnum* WC of the five *Sphagnum* species significantly differed between sites and seasons (LME; receptor site × season, *F*_7,261_ = 18.16, *P* < 0.0001, Fig. S6; Table S7), while no differences were found when species, receptor site, and season were combined (LME species × receptor site × season, *F*_7,230_ = 0.6, *P* = 0.96). In spring, the *Sphagnum* WC did not significantly differ across sites and among species with an overall mean of 15.8 ± 1.3 g H_2_O g^−1^ DW (species × receptor site, *F*_16,95_ = 1.3, *P* = 0.23). In summer, the WC of all *Sphagnum* species transplanted in Sweden (6.4 ± 0.34 3 g H_2_O g^−1^ DW) and Poland (7.5 ± 0.16 3 g H_2_O g^−1^ DW) was the lowest, and the highest in France (13.8 ± 0.8 g H_2_O g^−1^ DW) and Estonia (13 ±1.2 g H_2_O g^−1^ DW, receptor site, *F*_4,73_ = 16.4, *P* < 0.0001). In autumn, the highest *Sphagnum* WC was found in Poland (on average 24.7 ± 1.5 g H_2_O g^−1^ DW, *F*_3,44_ = 5.6, *P* = 0.002), while the average WC for the other sites was 19 ± 1.1 g H_2_O g^−1^ DW for all species.

Water‐extractable organic carbon and nitrogen (WEOC and WEON, respectively) contents significantly varied between seasons and across the five sites (LME season × receptor, WEOC: *F*_7,231_ = 10, *P* < 0.0001; WEON: *F*_7,231_ = 13, *P* < 0.0001; Figs S7, S8; Table S7). In spring, WEOC and WEON were the lowest in all *Sphagnum* species transplanted to France (on average 1.3 ± 0.05 mg C mg^−1^ DW and 0.04 ± 0.004 mg N mg^−1^ DW, respectively; WEOC: receptor site, *F*_4,95_ = 27.4, *P* < 0.0001; WEON: *F*_4,95_ = 14.4, *P* < 0.0001, LME). The highest WEOC was found in Poland (on average 5.7 ± 0.8 mg C mg^−1^ DW) and the highest WEON in Estonia (on average 0.2 ±0.03 mg N mg^−1^ DW). In summer, WEOC content was particularly high in France, Poland and Sweden for all *Sphagnum* species (Fig. S6, receptor site, *F*_4,72_ = 10.6, *P* < 0.0001), while WEON was the highest in France for all species (Fig. S7, receptor site, *F*_4,72_ = 11.2, *P* < 0.0001). In autumn, WEOC varied significantly between receptor sites (average of 7.42 ± 0.6 mg C mg^−1^ DW, receptor site, *F*_3,44_ = 2.9, *P* = 0.04), while WEON did not vary significantly between receptor sites (0.26 ± 0.02 mg N mg^−1^ DW, *P* > 0.05, LME). All statistical details about these results are provided in Table S7.

### Seasonal and transplantation effects on *Sphagnum* metabolites

**Fig. 2:**
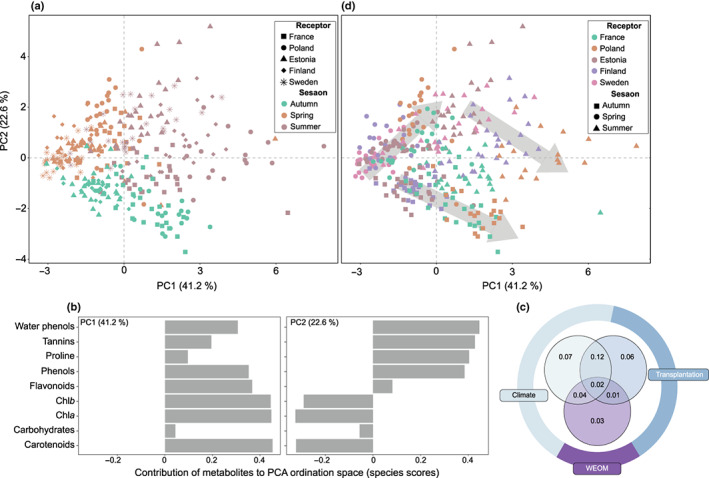
Principal component analysis (PCA) of standardized metabolites data collected along the latitudinal gradient over three seasons for (a) receptor sites presented as shapes and seasons as colour and (d) receptor sites presented as colours and seasons as shapes. Grey arrows indicate the north‐south transplant gradient for each season. (b) The loadings of each metabolite on the two PCA axes. (c) Variance partition modelling of the effects of climate (mean temperature and cumulated precipitation), transplantation (∆temperature and ∆precipitation) and WEOM (WEOC and WEON) on the *Sphagnum* metabolite composition (*n* = 375; adjusted *R* ^2^: 0.35). Numbers describe the proportion of variation in *Sphagnum* metabolites associated with each section of the diagram. The colours of the donut chart reflect the proportion of variation attributed to different categories, excluding variation shared by all three.

Regardless of *Sphagnum* species, the PCA evidenced a net seasonal effect on the *Sphagnum* metabolite composition (Fig. 2a). Every *Sphagnum* metabolite strongly varied across seasons in a similar direction for each *Sphagnum* species (Figs S9–S17; Table S7). In particular, our findings showed greater concentrations of primary (i.e. proline, Chl*a*, Chl*b* and carotenoids) and secondary metabolites (i.e. total tannins, phenols, flavonoids and water‐soluble phenols) in summer (Figs 2b, S9–S17). Redundancy analysis further revealed that seasonal variations of metabolites were strongly related to temperature and precipitation changes (Fig. S18; Table S8), which together explained 25% of total variation (Fig. 2c). WEOC and WEON were also important determinants of *Sphagnum* metabolites' seasonal variations, but to a lesser extent than temperature and precipitation changes (9.6% of variance explained, Fig. 2c). As strongly colinear with air temperature (*vif.cca* value > 10), PAR has been excluded from all RDA.

**Fig. 3:**
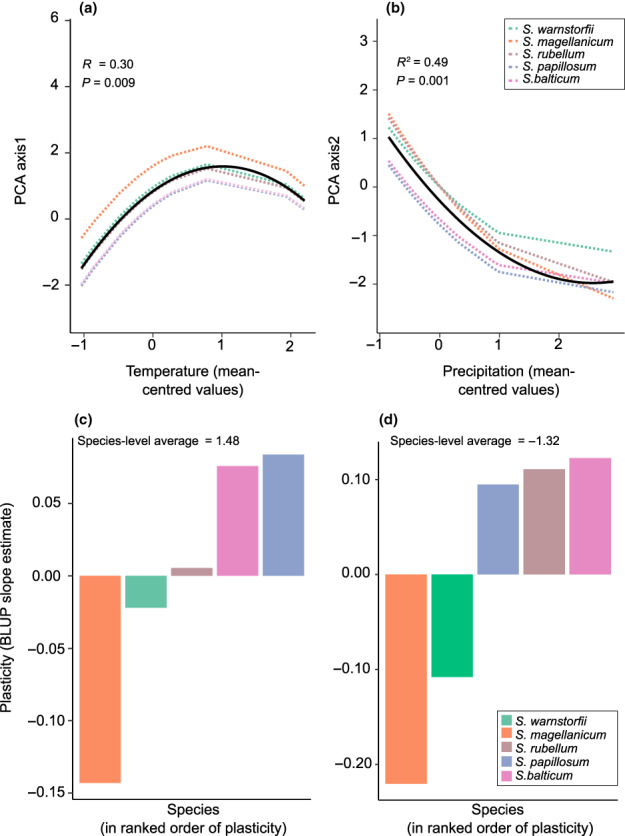
Metabolic plasticity across mean‐centred cumulative temperature (a) and precipitation (b) of the receptor sites for the five *Sphagnum* species over three seasons. The thick black line represents the quadratic regression model fit of the overall effect of temperature or precipitation (the predicted average of five *Sphagnum* species) on *Sphagnum* metabolic composition, and dashed coloured lines represent each *Sphagnum* species modelled reaction norms from the random regression mixed‐effects models (RRMM) that accounts for differences in intercept and slope (a, b) and quadratic curvature (b). Ranking *Sphagnum* species in order of plasticity estimated as best linear unbiased predictors (BLUP) slope estimates (c, d). More positive BLUP slope estimates represent greater plasticity (according to the predicted average of the five *Sphagnum* species), while negative BLUP slope estimates represent lower plasticity in the metabolite composition of *Sphagnum* mosses in response to mean‐centred temperatures (c). Negative BLUP slope estimates represent greater plasticity (according to the predicted average of the five *Sphagnum* species), while positive BLUP slope estimates represent lower plasticity in the metabolite composition of *Sphagnum* mosses in response to mean‐centred temperatures (d).

In addition to seasonal variations, the PCA showed a clear transplantation effect on the *Sphagnum* metabolite composition within seasons (Fig. 2d). The RDA (Fig. S18) showed that the transplant effect was significantly driven by variations of temperature (∆temperature) and precipitation (∆precipitation) between sites, which together explained 21% of total metabolite variation across seasons (Fig. 2c; Table S9). In particular, RRMM models between the *Sphagnum* metabolite composition (i.e. PCA scores on axes 1 and 2) and temperature or precipitation changes within sites and across seasons showed that all *Sphagnum* species had similar responses to climate change (i.e. similar reaction norms based on the slope and curvature of the curves; Fig. 3; Tables S5, S6). This finding was further corroborated by LME models performed on individual metabolites (Table S8). Overall, shifts in the *Sphagnum* metabolite composition on PCA axis 1 showed a positive relationship with increasing temperature over three seasons (*R* ^2^ = 0.30, *P* = 0.009; Fig. 3a; Table S5) as well as for each season (Fig. S19a–c). *Sphagnum balticum* and *S. papillosum* exhibited the highest degree of metabolite plasticity towards temperature change (BLUPs, Fig. 3b). RRMM models between PCA axes 1 of metabolites and precipitation change were not significant. RRMM models based on the relationship between *Sphagnum* metabolites variations on PCA axis 2 and precipitation change showed negative correlations over three seasons (*R* ^2^ = 0.49, *P* = 0.001; Fig. 3b; Table S6) and for every season (Fig. S19d–f). *Sphagnum magellanicum* and *S. rubellum* showed the highest degree of metabolite plasticity along with changing precipitation (Fig. 3b). RRMM models between PCA axes 2 of metabolites and temperature change were not significant.

**Fig. 4:**
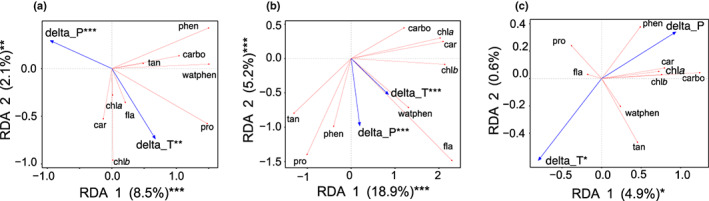
Climate change effects on *Sphagnum* metabolic composition across seasons. (a–c) Redundancy analysis (RDA) biplots in spring (a), summer (b) and autumn (c) (*n* = 125 per season). *Sphagnum* metabolite data have been standardized beforehand and constrained by temperature (∆temperature) and precipitation (∆precipitation) change across the transplant gradient. Metabolites are represented by red arrows and explanatory variables by blue arrows. Asterisks on axes and explanatory variables indicate whether they are or not significant at: *, *P* < 0.05; **, *P* < 0.01; ***, *P* < 0.001, respectively (ANOVAs). car, carotenoids; carbo, carbohydrates; delta_P, ∆precipitation between the reference site (Abisko) and the others; delta_T, ∆temperature between the reference site (Abisko) and the others; fla, flavonoids; phen, phenols; pro, proline; tan, tannins; watphen, water‐soluble phenolics.

The RDA performed for each season separately showed that different sets of compounds responded to temperature and precipitation changes within seasons (∆temperature and ∆precipitation, respectively; Fig. 4). In spring, all *Sphagnum* species transplanted to warmer conditions showed higher concentrations of pigments (carotenoids, Chl*a* and Chl*b*), flavonoids and proline than *Sphagnum* species under cold conditions (Fig. 4a). Wetter conditions promoted secondary metabolites such as water‐soluble phenolics and total phenols. In summer, our results showed that *Sphagnum* species transplanted to warmer and wetter conditions showed higher concentrations of water‐soluble phenols, flavonoids, total phenols and tannins when compared to *Sphagnum* species under colder conditions (Fig. 4b). They also showed lower pigments and carbohydrates but to a lesser extent. In autumn, all *Sphagnum* species transplanted in a warmer but drier climate systematically showed lower concentrations of pigments and higher concentrations of secondary metabolites such as tannins, than species under colder and wetter conditions (Fig. 4c).

### Seasonal patterns of gross ecosystem productivity and response to transplantation

**Fig. 5:**
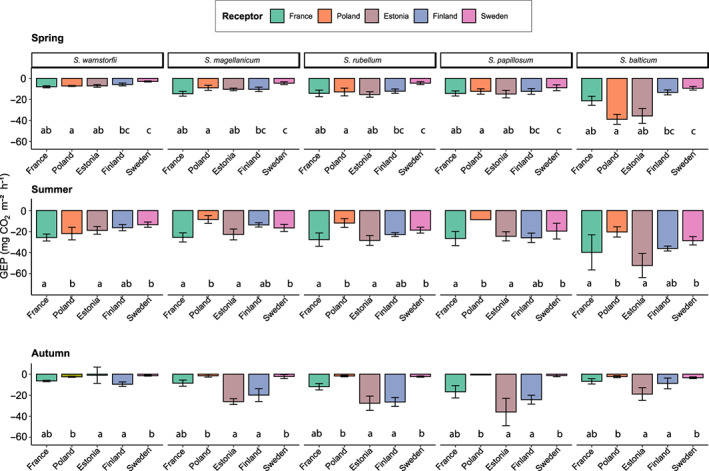
Gross ecosystem productivity (GEP, mg C m^−2^ h^−1^), standardized by vascular plant cover, in transplanted peat‐mesocosms over three seasons. Receptor sites are coloured, and dominant *Sphagnum* species are ordered according to the south‐north gradient. Each value represents mean ± SE (*n* = 5 independent plots per species and per site). Letters indicate significant differences between sites at *P* < 0.05 (linear mixed‐effects models, ANOVAs).

As shown for *Sphagnum* metabolites, GEP evidenced net seasonal patterns (LME, season, *F*_2,210_ = 39.2, *P* < 0.001; Fig. 5) with an increase in C uptake at the peak of the growing season (summer) in each site. Besides seasonal variations, GEP also exhibited transplantation effects although this effect was season‐dependent (Fig. 5; receptor site × season, *F*_7,208_ = 8.9, *P* < 0.001). In spring, all peat‐mesocosms transplanted to a warmer climate showed higher C uptake (on average GEP = −14.7 ±1.1 mg CO_2_ m^−2^ h^−1^; LME, ∆temperature, *F*_1,112_ = 4.8, *P* = 0.03, Table S9) than peat‐mesocosms under colder conditions and/or high diurnal range (on average −6.1 ± 0.9 mg CO_2_ m^−2^ h^−1^). In summer and autumn, GEP increased along with temperature increase but only under relatively wet conditions (summer: LME, ∆temperature × ∆precipitation, *F*_1,96_ = 5.3, *P* = 0.02; autumn: LME, ∆temperature × ∆precipitation, *F*_1,96_ = 41, *P* < 0.001, Table S9; Fig. 5). NEE and ER also showed strong seasonal patterns and responses to transplantation and are both detailed in the Supporting Information (Figs S20, S21; Table S7).

**Fig. 6:**
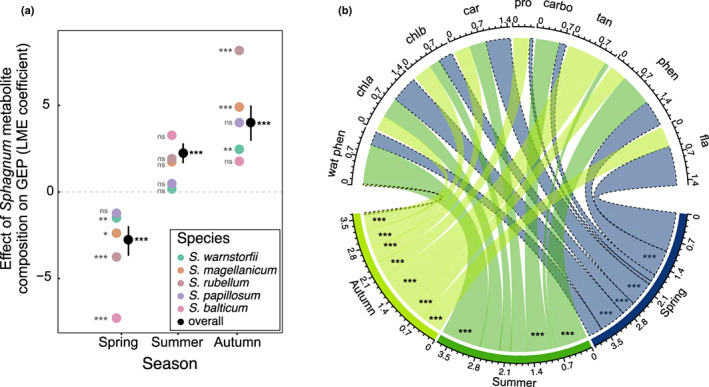
Effects of the *Sphagnum* metabolite composition on gross ecosystem productivity (GEP) across seasons. (a) Effects of *Sphagnum* metabolic composition (PCA axis 1) on GEP in spring, summer and autumn. Responses represent estimates ± SE from linear mixed‐effects (LME) models (black dots), with coloured points indicating the individual response of each species (SE has been removed on the individual responses for the sake of clarity of the figure). Points situated above or below zero (dotted line) indicate a positive or a negative relationship between *Sphagnum* metabolic composition and GEP, respectively. Detailed linear relationships are given in Fig. S21. (b) Correlations between individual *Sphagnum* metabolites and GEP. Edge width corresponds to the absolute value of the correlation coefficient determined by LMEs (i.e. estimates). Colours indicate the season, while dashed lines denote negative relationships between metabolites and GEP. Asterisks indicate significant correlations, with ***, *P* < 0.001 (ANOVAs). car, carotenoids; carbo, carbohydrates; fla, flavonoids; phen, phenols; pro, proline; tan, tannins; wat phen, water‐soluble phenolics.

In addition to ∆temperature and ∆precipitation, we found that shifts in GEP along the transplantation gradient were significantly related to changes in the *Sphagnum* metabolite composition across seasons (Figs 6a, S22). Similar patterns were found with NEE, while ER poorly responded to shifts in the *Sphagnum* metabolite composition across seasons (Figs S23, S24). This indicates that the linkages between NEE and the *Sphagnum* metabolite composition rather reflect the response of GEP, not ER. The overall relationship between GEP (and NEE) and the *Sphagnum* metabolite composition was significant with the first PCA axis of metabolites (LME, *F*_1,306_ = 4.39, *P* = 0.04), but not with the second PCA axis (LME, *F*_1,306_ = 2.63, *P* = 0.11, Table S10). Our findings further showed that the strength and direction of the relationship between the shifts in metabolites (PCA axis 1) and GEP (or NEE) varied with the season (Figs 6a, S23). In spring, GEP was negatively correlated with changes in metabolite composition (PCA axis 1), while in summer and autumn, the relationship was positive (Fig. 6a). In other words, GEP increased along with increasing concentrations of pigments, flavonoids and total phenols in spring and decreased with the rise of secondary metabolites (phenols, flavonoids and tannins) and carbohydrates in summer and autumn (Fig. 6b).

## Discussion

Our aim was to explore whether climate change drives metabolic plasticity in *Sphagnum* mosses, with cascading effects on peatland CO_2_ uptake. By performing a fully reciprocal peat transplant experiment across a gradient of nearly 10°C air temperature and 200 mm precipitation, we were able to expand upon previous findings to show that warming influences not only *Sphagnum* polyphenols (Jassey *et al*., 2011b) but also many primary and secondary metabolites. We find that *Sphagnum* species produce different concentrations of metabolites even when exposed to the same climate conditions at a site. However, the responses of the different species were parallel (Fig. 3); in other words, the production of metabolites of *Sphagnum* species changed in proportion to one another across the temperature gradient. Moreover, we find that a warmer climate caused plasticity in the seasonality of *Sphagnum* metabolites, increasing or lowering their investment in photosynthesis machinery depending on seasonal climatic conditions. *Sphagnum* mosses responded to warming with an upregulation of their primary metabolism in spring and an upregulation of their secondary metabolism in summer and autumn. While our results corroborate recent findings on the sensitivity of peatland net CO_2_ exchange to warming across seasons (Helbig *et al*., 2022), they also provide a mechanistic and physiological understanding of why spring warmth increases net CO_2_ uptake and summer and autumn warming lead to decreased net CO_2_ uptake. These results are the first demonstration that seasonality and plastic responses of *Sphagnum* metabolites to warming have the capacity to affect rates of ecosystem‐level processes involved in the peatland C cycle.

All *Sphagnum* species showed similar seasonal patterns, with the maximum concentrations of all metabolites but carbohydrates in summer. This result corroborates previous findings on polyphenols and flavonoids from *Sphagnum* (Chiapusio *et al*., 2018; Klavina *et al*., 2018) and feather mosses (Lunić *et al*., 2022). We interpret this finding as a physiological response to a more intense biological activity during summer (Lambers *et al*., 2008; Rousk *et al*., 2017; Thakur & Kapila, 2017). For instance, the high concentrations of pigments in summer (i.e. Chl*a* and Chl*b* and carotenoids) are directly related to the rise of GEP, indicating that increasing temperature promotes *Sphagnum* photosynthesis by supporting its photosynthetic machinery (Haraguchi & Yamada, 2011; Rastogi *et al*., 2020). In addition to primary metabolites, secondary metabolites increased in summer. This suggests that *Sphagnum* produces these compounds to cope with the abiotic stresses occurring in summer, such as high temperature, light irradiation and/or droughts (Iason *et al*., 2012; Gargallo‐Garriga *et al*., 2015; Sytiuk *et al*., 2022). For example, the increase in flavonoids in *Sphagnum* mosses, which was concomitant with the decrease in the *Sphagnum* WC, indicates that *Sphagnum* may have produced these compounds as an antioxidant to limit the oxidative stress resulting from drier conditions in summer (Choudhury *et al*., 2013; Das & Roychoudhury, 2014; Noctor *et al*., 2018). Besides environmental stress, the increase in secondary metabolites, such as water‐soluble phenolics, may further indicate that *Sphagnum* mosses were actively defending against herbivores (Chen *et al*., 2021) and/or vascular plants during summer (Chiapusio *et al*., 2013; Whitehead *et al*., 2018) through allelopathic effects. Finally, the low concentrations of carbohydrates in summer, but high in spring and autumn, could be explained by the seasonality in resource allocation of photosynthates (Hájek, 2014). At the end of the growing season, photosynthates can be stored in the *Sphagnum* capitula in the form of carbohydrates and thus provide cellular osmotic protection against freezing in winter (Skre *et al*., 1983). These findings show that the C assimilated by *Sphagnum* over seasons is allocated not only to growth and energy supply (increased pigments) but also to defensive mechanisms (increased secondary metabolites) regardless of *Sphagnum* species, providing mechanistic insight into *Sphagnum* ecology across seasons.

The reciprocal transplant experiment allowed us to test for the effects of temperature and precipitation changes on the *Sphagnum* metabolite composition and peatland C uptake and to distinguish between responses acting *via* plasticity and those acting *via* taxonomic differentiation. We found that the *Sphagnum* metabolite composition clearly responded to the transplant gradient. In particular, our findings evidenced that warming shifted *Sphagnum* metabolites composition over the seasons. Surprisingly, all *Sphagnum* species were chemically similar along the climate gradient for every season, with arctic‐originating species producing as many metabolites in the temperate environment as temperate‐originating species and vice versa. In other words, the *Sphagnum* metabolite composition is very plastic and tends to switch towards local optima when moved to new conditions, suggesting the advantage to adopt a similar phenotype to the home species to maximize fitness (Enquist *et al*., 2015; Muscarella & Uriarte, 2016). These findings thus emphasize processes that constrain the local *Sphagnum* metabolite composition, such as broad‐scale environmental filtering and fine‐scale niche partitioning (Muscarella & Uriarte, 2016). Our results are further consistent with the predictions that broad classes of plant metabolites are evolutionarily labile (Moreira *et al*., 2018; Yonekura‐Sakakibara *et al*., 2019; Defossez *et al*., 2021), adding support to the notion that plants can quickly adapt in the face of rapid climate change (Hairston *et al*., 2005; Walker *et al*., 2019).

The response of the *Sphagnum* metabolite composition to increasing temperatures varied across seasons. We found that *Sphagnum* invested more in primary metabolites such as Chl*a* and Chl*b* and carotenoids with spring warmth (Fig. 4a). This suggested that *Sphagnum* was photosynthetically more active (Rastogi *et al*., 2020). Spring warmth may have stimulated enzymes (e.g. RuBisCO) involved in photosynthetic pigment production (Andersson & Backlund, 2008). Indeed, the increase in chlorophyll content in *Sphagnum* tissues was concomitant with the decrease in available nitrogen in the interstitial *Sphagnum* water (Fig. S7), indicating that *Sphagnum* was taking up nitrogen. As RuBisCO is a nitrogen‐rich enzyme (Andersson & Backlund, 2008), we interpret these findings as evidence that spring warmth stimulated the RuBisCO pathway in *Sphagnum*, increasing pigment contents in tissues, and hence photosynthesis (Björkman, 1981). This was further confirmed by data, showing that increasing pigment content in *Sphagnum* tissues in spring was related to increasing GEP (Fig. 6). Alleviation from cold stress in spring may thus select *Sphagnum* phenotypes that produce more primary metabolites, reflecting a trade‐off between investment in growth and tissue longevity (Díaz *et al*., 2016). However, this trade‐off in favour of growth reversed with summer warming as *Sphagnum* invested more in secondary metabolites – particularly phenols and flavonoids (Fig. 4b). The increase in these compounds with summer warming suggests that *Sphagnum* was probably stressed by high temperatures (Laoué *et al*., 2022). For example, several studies conducted on Mediterranean species evidenced an increase in the production of flavonoids and phenols when plants suffer the most from high temperatures (Vogt *et al*., 1987; Chaves *et al*., 1993, 1997; Laoué *et al*., 2022). These findings suggest that *Sphagnum* mosses invested more in defence during summer warming to cope with high temperatures, and they did so at the expense of photosynthesis, as evidenced by the negative effect of increasing secondary metabolites on GEP in summer (Fig. 6). In autumn, *Sphagnum* used different metabolic mechanisms as those involved with spring and summer warming. On the opposite to what one would have expected, autumn warming did not prolong *Sphagnum* productivity by slowing chlorophyll degradation (Shi *et al*., 2014) but increased carbohydrate accumulation in tissues instead. This indicates that photosynthates were stored in the form of carbohydrates and not chlorophyll to support the development of cold hardiness (Angelcheva *et al*., 2014; Hájek & Vicherová, 2014) without suppressing the benefits of warming on photosynthesis. These results imply that warming profoundly influences the balance of *Sphagnum* life history across seasons, an effect that can subsequently shift net peatland CO_2_ uptake across seasons (Helbig *et al*., 2022).

*Sphagnum* exposure to a warmer climate caused metabolic plasticity that was related to increased CO_2_ uptake in the early and late growing seasons. We thus show that the plasticity of *Sphagnum* metabolic composition as a response to seasonality in warming trends may have the capacity to buffer, or even reverse, the negative effects of warming on peatland CO_2_ uptake during summer, although such an effect may depend on the region considered (Helbig *et al*., 2022). These findings demonstrate a clear potential for *Sphagnum* mosses to modify the magnitude and direction of feedback from ecosystems to future climate change, corroborating the most recent model simulation and predictions of the peatland C sink (Gallego‐Sala *et al*., 2018; Laine *et al*., 2019). However, the extent to which these metabolic mechanisms will occur depends on whether *Sphagnum* metabolic plasticity keeps pace with rapid climate change (Hairston *et al*., 2005), and whether other factors associated with temperature change, such as vascular plant encroachment (Chiapusio *et al*., 2018), become dominant selective forces. We caveat that we measured the *Sphagnum* metabolite composition and ecosystem CO_2_ uptake at local scales at only three dates in the growing season. Future high‐resolution studies are needed to test the generality of the responses detected here and their importance for peatland net CO_2_ exchanges over time. Nevertheless, our findings are supported by recent findings on peatland net CO_2_ exchanges from multiannual monitoring (Helbig *et al*., 2022), thus providing confidence in the mechanisms found here. Furthermore, we observed similar relationships between metabolic plasticity and GEP across five different species from different origins, suggesting that the relationship between the *Sphagnum* metabolite composition and GEP is consistent throughout the *Sphagnum* genus. Because temperature can cause metabolic plasticity in multiple vascular plant species (Peñuelas *et al*., 2013; Gargallo‐Garriga *et al*., 2020; Laoué *et al*., 2022), the possibility that the metabolic mechanisms discovered here occur in other peatland plant species exists, with potentially similar consequences for peatland net CO_2_ uptake. In summary, our findings illustrate the capacity of plants to adapt their chemistry in response to warming across seasons, with immediate effects on ecosystem C uptake, thus revealing a further mechanism by which plants influence ecosystem responses to climate change. To conclude, a concerted effort to continue observations of the linkages between plant metabolite plasticity and peatland CO_2_ exchanges across space and time at high resolution is urgently needed if we want to better understand future changes in the northern peatland CO_2_ sink.

## Competing interests

None declared.

## Author contributions

VEJJ conceived the experimental design with input from ED, EST, ML, MK and RC. SH and VEJJ performed the reciprocal transplantation with the help of RC, MK, ML, EST and ED. AS, SH and VEJJ performed fieldwork with the help of MK. AS and HG did the metabolite analyses. AS and VEJJ performed GEP measurements in the field, and AS calculated flux rates. AS and SH undertook laboratory work relating to WEOM analyses and *Sphagnum* WC measurements. AS and VEJJ analysed the data and wrote the manuscript in close consultation with RC and SH and with input from all co‐authors.

## Supporting information


**Fig. S1** *Sphagnum* length increment in peat‐mesocosms from France and Estonia 3 months after transplantation.
**Fig. S2** Temperature, total photosynthetically active radiation and precipitation variability in each site for every season.
**Fig. S3** Principal component analysis on *Sphagnum* metabolites over the three seasons in the plastic boxes (which stayed at their site of origin) and at the control plots on the sites (untouched plots without boxes).
**Fig. S4** Dynamic of gross ecosystem productivity quantified over three seasons in the boxes (which stayed at their site of origin) and at the control plots on the sites (untouched plots without box).
**Fig. S5** pH values in the plastic boxes from the same site dispatched along the gradient.
**Fig. S6** Barplot of *Sphagnum* water content in the transplanted mesocosms for each species across seasons and sites.
**Fig. S7** Barplot of water‐extractable organic carbon of each *Sphagnum* species collected in the transplanted mesocosms across seasons and sites.
**Fig. S8** Barplot of water‐extractable organic nitrogen of each *Sphagnum* species collected in the transplanted mesocosms across seasons and sites.
**Fig. S9** Chl*a* content in *Sphagnum* tissues collected in the transplanted mesocosms across seasons and sites.
**Fig. S10** Chl*b* content in *Sphagnum* tissues collected in the transplanted mesocosms across seasons and sites.
**Fig. S11** Carotenoid content in *Sphagnum* tissues collected in the transplanted mesocosms across seasons and sites.
**Fig. S12** Carbohydrates content in *Sphagnum* tissues collected in the transplanted mesocosms across seasons and sites.
**Fig. S13** Water‐soluble phenolic content in *Sphagnum* tissues collected in the transplanted mesocosms across seasons and sites.
**Fig. S14** Proline content in *Sphagnum* tissues collected in the transplanted mesocosms across seasons and sites.
**Fig. S15** Tannin content in *Sphagnum* tissues collected in the transplanted mesocosms across seasons and sites.
**Fig. S16** Total phenolic content in *Sphagnum* tissues collected in the transplanted mesocosms across seasons and sites.
**Fig. S17** Flavonoid content in *Sphagnum* tissues collected in the transplanted mesocosms across seasons and sites.
**Fig. S18** Redundancy analysis of metabolites in transplanted *Sphagnum* species over three seasons in relation to local (water‐extractable organic carbon and water‐extractable organic nitrogen) and regional (temperature and precipitation) environmental variables.
**Fig. S19** Metabolic plasticity across mean‐centred cumulative temperatures and precipitation of the receptor sites for the five *Sphagnum* species in spring, summer and autumn.
**Fig. S20** Net ecosystem exchange (mg C m^−2^ h^−1^), standardized by vascular plant cover, in transplanted peat‐mesocosms over three seasons.
**Fig. S21** Ecosystem respiration (mg C m^−2^ h^−1^), standardized by vascular plant cover, in transplanted peat‐mesocosms over three seasons.
**Fig. S22** Correlation between the *Sphagnum* metabolite composition (PCA axis 1) and gross ecosystem productivity (standardized values) in transplanted peat‐mesocosms across seasons.
**Fig. S23** Correlation between the *Sphagnum* metabolite composition (PCA axis 1) and net ecosystem exchange (standardized values) in transplanted peat‐mesocosms across seasons.
**Fig. S24** Correlation between the *Sphagnum* metabolite composition (PCA axis 1) and ecosystem respiration (standardized values) in transplanted peat‐mesocosms across seasons.
**Table S1** Summary of the climatic data taken in each of the five sites.
**Table S2** Summary of pairwise comparison of *Sphagnum* metabolites among samples collected in the plastic boxes (boxes that stayed at the site of their origin) and outside of the box (untouched plot) over the three seasons.
**Table S3** Summary of pairwise comparison of *Sphagnum* gross ecosystem productivity measurements in the plastic boxes (boxes that stayed at the site of their origin) and outside of the boxes (untouched plots) over the three seasons.
**Table S4** Summary of linear mixed‐effects models testing the receptor site effect (fixed effect) on transplanted box pH.
**Table S5** Summary of random regression mixed models with PC1 on metabolites as the response variable.
**Table S6** Summary of random regression mixed models with PC2 on metabolites as the response variable.
**Table S7** Summary of linear mixed‐effects models testing the effect of receptor site, species and season (fixed effects) on *Sphagnum* water content, water‐extractable organic matter, water‐extractable organic carbon, gross ecosystem productivity and *Sphagnum* metabolites.
**Table S8** Summary of linear mixed‐effects models testing the effect of mean temperature and precipitation (fixed effects) on *Sphagnum* water content, water‐extractable organic matter, water‐extractable organic carbon, gross ecosystem productivity and *Sphagnum* metabolites.
**Table S9** Summary of linear mixed‐effects models (*lmer*) testing the effect of delta temperature and delta precipitation (fixed effects) on *Sphagnum* water content, water‐extractable organic matter, water‐extractable organic carbon, gross ecosystem productivity and *Sphagnum* metabolites.
**Table S10** Summary of linear models testing the correlation between gross ecosystem productivity (fixed effect) and PC1 and PC2 on metabolites.
Please note: Wiley is not responsible for the content or functionality of any Supporting Information supplied by the authors. Any queries (other than missing material) should be directed to the *New Phytologist* Central Office.

## Data Availability

Data related to this paper are available from Figshare (doi: 10.6084/m9.figshare.c.6278640.v1).
