# Supplementary material for: Linkages between Sphagnum metabolites and peatland CO2 uptake are sensitive to seasonality in warming trends
Source: New Phytol. 2022 Dec 7;237(4):1164–78. doi: 10.1111/nph.18601 (PMC10108112; doi:10.1111/nph.18601)
Supplement: Supplementary file 1 — Fig. S1 Sphagnum length increment in peat‐mesocosms from France and Estonia 3 months after transplantation. Fig. S2 Temperature, total photosynthetically active radiation and precipitation variability in each site for every season. Fig. S3 Principal component analysis on Sphagnum metabolites over the three seasons in the plastic boxes (which stayed at their site of origin) and at the control plots on the sites (untouched plots without boxes). Fig. S4 Dynamic of gross ecosystem productivity quantified over three seasons in the boxes (which stayed at their site of origin) and at the control plots on the sites (untouched plots without box). Fig. S5 pH values in the plastic boxes from the same site dispatched along the gradient. Fig. S6 Barplot of Sphagnum water content in the transplanted mesocosms for each species across seasons and sites. Fig. S7 Barplot of water‐extractable organic carbon of each Sphagnum species collected in the transplanted mesocosms across seasons and sites. Fig. S8 Barplot of water‐extractable organic nitrogen of each Sphagnum species collected in the transplanted mesocosms across seasons and sites. Fig. S9 Chla content in Sphagnum tissues collected in the transplanted mesocosms across seasons and sites. Fig. S10 Chlb content in Sphagnum tissues collected in the transplanted mesocosms across seasons and sites. Fig. S11 Carotenoid content in Sphagnum tissues collected in the transplanted mesocosms across seasons and sites. Fig. S12 Carbohydrates content in Sphagnum tissues collected in the transplanted mesocosms across seasons and sites. Fig. S13 Water‐soluble phenolic content in Sphagnum tissues collected in the transplanted mesocosms across seasons and sites. Fig. S14 Proline content in Sphagnum tissues collected in the transplanted mesocosms across seasons and sites. Fig. S15 Tannin content in Sphagnum tissues collected in the transplanted mesocosms across seasons and sites. Fig. S16 Total phenolic content in Sphagnum tissues collected in the [file NPH-237-1164-s001.pdf]

New Phytologist Supplementary Information

Linkages between *Sphagnum* metabolites and peatland CO_2 uptake are sensitive to seasonality in warming trends

Anna Sytiuk, Samuel Hamard, Regis Céréghino, Ellen Dorrepaal, Honorine Geissel, Martin Küttim, Mariusz Lamentowicz, Eeva-Stiina Tuittila, Vincent E.J. Jassey

Article acceptance: 31 October 2022

This file contains:

- Supplementary Tables S1 – S10
- Supplementary Figures S1 – S24

SUPPLEMENTARY TABLES

Table S1. Climatic data for the five study sites 30 days before sampling. Cumulative temperature and precipitation were calculated as summed values per every season of 2019. Δtemperature and Δprecipitation were calculated by extracting the mean temperature and precipitation, respectively, of the coldest site (Sweden, as a reference site) from the other four sites (the seasonality of the transplantation effect). Temperature and precipitation seasonality were calculated on averaged data over the period 1960-2018 (Fick & Hijmans, 2017).

country	season	mean temperature, °C	precipitation, mm	PAR, µmol photons/m²/day	cumulative temperature	cumulative diurnal range	temperature seasonality	precipitation seasonality	Δ temperature	Δ precipitation
France	spring	4.8	92	7847	151	194	54.5	1.5	3.5	-36.6
Poland	spring	6.0	11	6890	192	289	78.5	2.7	4.7	-117.6
Estonia	spring	8.1	40	9484	252	321	84.7	3.1	6.8	-88.6
Finland	spring	6.8	31	7868	214	275	86.0	3.1	5.5	-97.6
Sweden	spring	1.3	129	8391	42	141	75.2	3.8	0	0
France	summer	15.2	37	8635	957	253	54.5	1.5	4.1	8
Poland	summer	19.8	60	13320	1306	291	78.5	2.7	8.6	31
Estonia	summer	15.8	69	10523	1280	254	84.7	3.1	4.6	40
Finland	summer	14.3	53	9433	1149	295	86.0	3.1	3.2	24
Sweden	summer	11.1	29	9555	670	210	75.2	3.8	0	0
France	autumn	2.9	210	2010	3668	213	54.5	1.5	-2.4	146
Poland	autumn	8.9	68	2646	3367	180	78.5	2.7	3.6	4
Estonia	autumn	7.9	105	4256	2082	237	84.7	3.1	2.6	41
Finland	autumn	7.4	73	3068	1512	197	86.0	3.1	2.1	9
Sweden	autumn	5.3	64	2980	1349	126	75.2	3.8	0	0

Table S2. Summary of pairwise comparison of *Sphagnum* metabolites among samples collected in the plastic boxes (boxes that stayed at the site of their origin) and outside of the box (untouched plot) over the three seasons. Asterisks indicate and values in bold indicate significant effects of plastic box at $P < 0.05$, ns = non-signficant.

Season	metabolite	country	group1	group2	n1	n2	statistic	df	P	P. significance
autumn	carotenoids	Estonia	in the box	on the site	5	5	-1.320595043	7.961450049	0.223	ns
autumn	carotenoids	Finland	in the box	on the site	5	5	0.592062237	7.999917996	0.57	ns
autumn	carotenoids	France	in the box	on the site	5	5	1.040921372	6.585305013	0.335	ns
autumn	carotenoids	Poland	in the box	on the site	4	5	-0.169363099	6.403937285	0.871	ns
autumn	carbohydrates	Estonia	in the box	on the site	5	5	1.685135824	5.452236511	0.148	ns
autumn	carbohydrates	Finland	in the box	on the site	5	5	-1.317130589	7.988914702	0.224	ns
autumn	carbohydrates	France	in the box	on the site	5	5	0.841328486	5.623209229	0.434	ns
autumn	carbohydrates	Poland	in the box	on the site	4	5	-3.067682863	4.627860893	***0.0308***	*
autumn	chlorophyll a	Estonia	in the box	on the site	5	5	-1.273594418	7.82086972	0.239	ns
autumn	chlorophyll a	Finland	in the box	on the site	5	5	0.586100825	7.991690692	0.574	ns
autumn	chlorophyll a	France	in the box	on the site	5	5	1.084109743	6.705965861	0.316	ns
autumn	chlorophyll a	Poland	in the box	on the site	4	5	0.133920038	6.997149993	0.897	ns
autumn	chlorophyll b	Estonia	in the box	on the site	5	5	-1.728463168	7.376268295	0.125	ns
autumn	chlorophyll b	Finland	in the box	on the site	5	5	0.668146612	7.999171717	0.523	ns
autumn	chlorophyll b	France	in the box	on the site	5	5	1.023337147	5.077683132	0.352	ns
autumn	chlorophyll b	Poland	in the box	on the site	4	5	-1.124190125	4.063389523	0.323	ns
autumn	flavonoids	Estonia	in the box	on the site	5	5	2.600473328	5.683143052	***0.0427***	*
autumn	flavonoids	Finland	in the box	on the site	5	5	1.405634375	7.704668924	0.199	ns
autumn	flavonoids	France	in the box	on the site	5	5	-0.876832226	6.895069459	0.41	ns
autumn	flavonoids	Poland	in the box	on the site	4	5	0.324132355	6.080436847	0.757	ns

autumn	total phenols	Estonia	in the box	on the site	5	5	0.45658641	4.29635879	0.67	ns
autumn	total phenols	Finland	in the box	on the site	5	5	0.747697812	7.998714875	0.476	ns
autumn	total phenols	France	in the box	on the site	5	5	-1.084412561	6.603396026	0.316	ns
autumn	total phenols	Poland	in the box	on the site	4	5	0.709232711	5.588138821	0.507	ns
autumn	proline	Estonia	in the box	on the site	5	5	-0.902766786	6.693261235	0.398	ns
autumn	proline	Finland	in the box	on the site	5	5	0.991753169	7.433135364	0.352	ns
autumn	proline	France	in the box	on the site	5	5	-0.997642292	6.469317563	0.354	ns
autumn	proline	Poland	in the box	on the site	4	5	0.650870762	4.660323714	0.546	ns
autumn	tannins	Estonia	in the box	on the site	5	5	-2.924383182	6.146649336	***0.0257***	*
autumn	tannins	Finland	in the box	on the site	5	5	0.554678487	6.525505272	0.598	ns
autumn	tannins	France	in the box	on the site	5	5	-0.831472911	5.751664874	0.439	ns
autumn	tannins	Poland	in the box	on the site	4	5	0.993539197	3.132026096	0.391	ns
autumn	water-soluble phenols	Estonia	in the box	on the site	5	5	0.601964059	7.468700881	0.565	ns
autumn	water-soluble phenols	Finland	in the box	on the site	5	5	-1.432689611	7.262705306	0.194	ns
autumn	water-soluble phenols	France	in the box	on the site	5	5	1.322715439	5.313711585	0.24	ns
autumn	water-soluble phenols	Poland	in the box	on the site	4	5	-1.988617433	3.312558357	0.132	ns
spring	carotenoids	Estonia	in the box	on the site	5	5	0.608272207	7.990118447	0.56	ns
spring	carotenoids	Finland	in the box	on the site	5	5	0.372815977	7.920863254	0.719	ns
spring	carotenoids	France	in the box	on the site	5	5	1.251736508	7.085202607	0.25	ns
spring	carotenoids	Poland	in the box	on the site	4	3	-11.53636857	2.200383485	***0.00521***	**
spring	carotenoids	Sweden	in the box	on the site	5	5	-0.17757547	5.562725376	0.865	ns
spring	carbohydrates	Estonia	in the box	on the site	5	5	1.550181702	4.649816576	0.186	ns

spring	carbohydrates	Finland	in the box	on the site	5	5	0.236938813	5.898484171	0.821	ns
spring	carbohydrates	France	in the box	on the site	5	5	3.809473814	7.680978108	***0.00557***	**
spring	carbohydrates	Poland	in the box	on the site	4	3	-7.424563687	4.844296256	***0.0008***	***
spring	carbohydrates	Sweden	in the box	on the site	5	5	0.598339149	7.370399152	0.568	ns
spring	chlorophyll a	Estonia	in the box	on the site	5	5	0.131688035	7.998923466	0.898	ns
spring	chlorophyll a	Finland	in the box	on the site	5	5	0.389100563	7.821537344	0.708	ns
spring	chlorophyll a	France	in the box	on the site	5	5	1.330406897	7.761087591	0.221	ns
spring	chlorophyll a	Poland	in the box	on the site	4	3	-12.62354389	2.486689447	0.00256	**
spring	chlorophyll a	Sweden	in the box	on the site	5	5	-0.913138263	6.819786449	0.392	ns
spring	chlorophyll b	Estonia	in the box	on the site	5	5	1.68296355	6.981476176	0.136	ns
spring	chlorophyll b	Finland	in the box	on the site	5	5	1.218985778	5.193268234	0.275	ns
spring	chlorophyll b	France	in the box	on the site	5	5	0.193115783	4.269771711	0.856	ns
spring	chlorophyll b	Poland	in the box	on the site	4	3	-15.1135555	2.209739168	***0.00284***	**
spring	chlorophyll b	Sweden	in the box	on the site	5	5	-0.512736538	6.288598667	0.626	ns
spring	flavonoids	Estonia	in the box	on the site	5	5	3.152803317	4.146260205	***0.0328***	*
spring	flavonoids	Finland	in the box	on the site	5	5	1.859707826	7.694274105	0.101	ns
spring	flavonoids	France	in the box	on the site	5	5	1.570163259	7.991364911	0.155	ns
spring	flavonoids	Poland	in the box	on the site	4	3	1.223417403	4.728659978	0.279	ns
spring	flavonoids	Sweden	in the box	on the site	5	5	-3.393442458	7.215366207	***0.011***	*
spring	total phenols	Estonia	in the box	on the site	5	5	1.827554152	4.30203878	0.137	ns
spring	total phenols	Finland	in the box	on the site	5	5	1.157216023	5.55821783	0.294	ns
spring	total phenols	France	in the box	on the site	5	5	3.771236861	5.134589725	***0.0124***	*

spring	total phenols	Poland	in the box	on the site	4	3	5.541122053	3.015594609	***0.0114***	*
spring	total phenols	Sweden	in the box	on the site	5	5	-0.644709666	7.085643877	0.539	ns
spring	proline	Estonia	in the box	on the site	5	5	-0.324236772	5.540432573	0.758	ns
spring	proline	Finland	in the box	on the site	5	5	1.484529459	7.983655783	0.176	ns
spring	proline	France	in the box	on the site	5	5	2.178185773	7.497968379	0.0633	ns
spring	proline	Poland	in the box	on the site	4	3	-0.546688282	4.424775985	0.611	ns
spring	proline	Sweden	in the box	on the site	5	5	-1.073552978	4.780748505	0.334	ns
spring	tannins	Estonia	in the box	on the site	5	5	-0.586606587	7.196715328	0.575	ns
spring	tannins	Finland	in the box	on the site	5	5	2.018151651	4.810591312	0.102	ns
spring	tannins	France	in the box	on the site	5	5	2.600887304	4.091473644	0.0587	ns
spring	tannins	Poland	in the box	on the site	4	3	1.179391327	4.219620029	0.3	ns
spring	tannins	Sweden	in the box	on the site	5	5	-1.802079938	7.416092272	0.112	ns
spring	water-soluble phenols	Estonia	in the box	on the site	5	5	0.572379616	4.08725922	0.597	ns
spring	water-soluble phenols	Finland	in the box	on the site	5	5	-0.79985946	4.659540751	0.463	ns
spring	water-soluble phenols	France	in the box	on the site	5	5	2.589524084	7.362337155	***0.0344***	*
spring	water-soluble phenols	Poland	in the box	on the site	4	3	0.111715875	4.970818758	0.915	ns
spring	water-soluble phenols	Sweden	in the box	on the site	5	5	1.607138063	7.056124755	0.152	ns
summer	carotenoids	Estonia	in the box	on the site	5	5	1.888307893	6.488592157	0.104	ns
summer	carotenoids	Finland	in the box	on the site	5	5	1.022952092	7.622669529	0.338	ns
summer	carotenoids	France	in the box	on the site	5	5	-1.071138107	7.940258362	0.316	ns
summer	carotenoids	Poland	in the box	on the site	4	5	-0.842503762	6.822589504	0.428	ns
summer	carotenoids	Sweden	in the box	on the site	5	5	-0.955771008	6.026941852	0.376	ns

summer	carbohydrates	Estonia	in the box	on the site	5	5	-0.954481943	6.55291863	0.374	ns
summer	carbohydrates	Finland	in the box	on the site	5	5	0.04150034	6.572369235	0.968	ns
summer	carbohydrates	France	in the box	on the site	5	5	3.369811885	6.916070083	***0.0121***	*
summer	carbohydrates	Poland	in the box	on the site	4	5	0.347389253	5.01902382	0.742	ns
summer	carbohydrates	Sweden	in the box	on the site	5	5	-0.331435162	7.97583943	0.749	ns
summer	chlorophyll a	Estonia	in the box	on the site	5	5	1.807842554	6.296445235	0.118	ns
summer	chlorophyll a	Finland	in the box	on the site	5	5	0.9579635	7.481824501	0.368	ns
summer	chlorophyll a	France	in the box	on the site	5	5	-1.099394506	7.641232252	0.305	ns
summer	chlorophyll a	Poland	in the box	on the site	4	5	-0.835583158	6.868163867	0.431	ns
summer	chlorophyll a	Sweden	in the box	on the site	5	5	-1.044955361	6.597519974	0.333	ns
summer	chlorophyll b	Estonia	in the box	on the site	5	5	2.324491281	6.06695761	0.0586	ns
summer	chlorophyll b	Finland	in the box	on the site	5	5	0.92487837	7.481643053	0.384	ns
summer	chlorophyll b	France	in the box	on the site	5	5	-0.226091438	7.869560912	0.827	ns
summer	chlorophyll b	Poland	in the box	on the site	4	5	-1.149479338	6.430127166	0.291	ns
summer	chlorophyll b	Sweden	in the box	on the site	5	5	-0.919095471	5.53691412	0.396	ns
summer	flavonoids	Estonia	in the box	on the site	5	5	-0.221519456	5.780499908	0.832	ns
summer	flavonoids	Finland	in the box	on the site	5	5	1.954718801	6.91078492	0.0921	ns
summer	flavonoids	France	in the box	on the site	5	5	-3.263966002	5.329161028	***0.0204***	*
summer	flavonoids	Poland	in the box	on the site	4	5	1.609485394	3.476876507	0.193	ns
summer	flavonoids	Sweden	in the box	on the site	5	5	5.38880787	7.661356889	***0.000757***	***
summer	total phenols	Estonia	in the box	on the site	5	5	2.195199829	5.557969265	0.0741	ns
summer	total phenols	Finland	in the box	on the site	5	5	0.848741128	7.768690055	0.421	ns

summer	total phenols	France	in the box	on the site	5	5	0.851305626	7.946693056	0.42	ns
summer	total phenols	Poland	in the box	on the site	4	5	-0.52074373	3.865482203	0.631	ns
summer	total phenols	Sweden	in the box	on the site	5	5	-1.031820521	7.035902924	0.336	ns
summer	proline	Estonia	in the box	on the site	5	5	2.97594355	5.086001276	***0.0303***	*
summer	proline	Finland	in the box	on the site	5	5	1.732904752	7.973859503	0.121	ns
summer	proline	France	in the box	on the site	5	5	0.744733375	5.341133847	0.488	ns
summer	proline	Poland	in the box	on the site	4	5	0.627201892	6.831684554	0.551	ns
summer	proline	Sweden	in the box	on the site	5	5	-0.975971051	4.355550756	0.38	ns
summer	tannins	Estonia	in the box	on the site	5	5	4.812906317	4.674491025	***0.00576***	**
summer	tannins	Finland	in the box	on the site	5	5	-1.695533853	4.962600941	0.151	ns
summer	tannins	France	in the box	on the site	5	5	3.384837372	5.51882568	***0.0168***	*
summer	tannins	Poland	in the box	on the site	4	5	1.153857204	6.273927566	0.291	ns
summer	tannins	Sweden	in the box	on the site	5	5	1.15257256	4.444766649	0.307	ns
summer	water-soluble phenols	Estonia	in the box	on the site	5	5	0.437520498	7.899310528	0.673	ns
summer	water-soluble phenols	Finland	in the box	on the site	5	5	1.073822545	5.905865651	0.325	ns
summer	water-soluble phenols	France	in the box	on the site	5	5	-0.755088646	4.603703009	0.487	ns
summer	water-soluble phenols	Poland	in the box	on the site	4	5	1.167862995	6.133167978	0.286	ns
summer	water-soluble phenols	Sweden	in the box	on the site	5	5	-0.370167531	7.820637198	0.721	ns

Table S3. Summary of pairwise comparison of *Sphagnum* gross ecosystem productivity (GEP) measurements in the plastic boxes (boxes that stayed at the site of their origin) and outside of the boxes (untouched plots) over the three seasons. Asterisks indicate a significant effect at $P < 0.05$; ns = non-significant.

Season	group1	group2	n1	n2	statistic	df	P	P significance
Autumn	in the box	on the site	19	25	0.0839	42.0	0.929	ns
Spring	in the box	on the site	24	23	-1.34	26.6	0.192	ns
Summer	in the box	on the site	24	25	-0.541	43.6	0.591	ns

Table S4. Summary of linear effect mixed models testing the receptor site effect (fixed effect) on transplanted box pH. Values in bold indicate a significant effect at $P < 0.05$.

Species	Df	*F*	*P*
All species	2, 39	1.04	0.36
S. balticum	2, 6	1.35	0.32
S. papillosum	2, 3	8.47	0.06
S. rubellum	2, 6	1.47	0.3
S. magellanicum	2, 6	0.35	0.71
S. warnstorfii	2, 6	0.26	0.78

Table S5. Summary of random regression mixed models (RRMMs) with PC1 on metabolites as the response variable and cumulated temperature as fixed variable. Values in bold indicate a significant effect at $P < 0.05$. The following models were tested: M1. Linear model with random effect (intercept) of (1|receptor), M2. Basic linear model with random effect (intercept) of (1|receptor) and (1|origin), M3. Basic linear model with random effect of origin varies both in intercept and slope (1|receptor)+ (1+receptor|orign), M4. Quadric fixed-effect model (quadric polynomial) with random effect (intercept) of (1|receptor), M5. Quadric fixed-effect model (quadric polynomial) with random effects (intercept) of (1|receptor) and (1|origin), M6. Quadric fixed-effect model (quadric polynomial) with random effect of origin vary both in intercept and slope (1|receptor)+ (1+receptor|orign), M7. Quadric fixed-effect model (quadric polynomial) with random effect of origin vary both in intercept and slope and curvature (1|receptor)+ (1+receptor^2|orign). The best model is highlighted italicized.

All seasons												
	M1			M2			M3			M4		
Predictors	Estimates	Statistic	p	Estimates	Statistic	p	Estimates	Statistic	p	Estimates	Statistic	p
(Intercept)	0.11	0.27	0.785	0.11	0.24	0.811	0.11	0.24	0.811	0.84	1.59	0.117
ctemperature	0.73	1.74	0.086	0.73	1.73	0.089	0.73	1.72	0.091			
ctemperature, 2 [1st degree]										1.48	2.74	**0.008**
ctemperature, 2 [2nd degree]										-0.73	-1.93	0.059
**Random Effects**												
σ^2	0.59			0.33			0.32			0.59		
τ_{00}	2.29 ctemperature			2.38 ctemperature<br>0.25 origin.x			2.39 ctemperature<br>0.25 origin.x			1.79 ctemperature		
τ_{11}							0.01 origin.x.ctemperature					
ρ_{01}							-1.00 origin.x					
ICC	0.79			0.89						0.75		
N	14 ctemperature			14 ctemperature<br>5 origin.x			14 ctemperature<br>5 origin.x			14 ctemperature		
Observations	70			70			70			70		
Marginal R^2 / Conditional R^2	0.155 / 0.827			0.152 / 0.906			0.152 / 0.909			0.304 / 0.827		
AIC	211.918			191.021			193.504			211.152		

log-Likelihood	-102.059	-90.75	-89.996	-100.414

	M5			M6			M7		
Predictors	Estimates	Statistic	p	Estimates	Statistic	p	Estimates	Statistic	p
(Intercept)	0.84	1.45	0.151	0.84	1.45	0.151	*0.84*	*1.34*	*0.184*
ctemperature									
ctemperature, 2 [1st degree]	1.48	2.72	**0.008**	1.48	2.71	0.009	*1.48*	*2.7*	***0.009***
ctemperature, 2 [2nd degree]	-0.73	-1.91	0.061	-0.73	-1.91	0.061	*-0.73*	*-1.84*	*0.071*
**Random Effects**									
σ^2	0.33			0.32			*0.26*		
τ_{00}	1.88 ctemperature			1.88 ctemperature			*1.90 ctemperature*		
	0.24 origin.x			0.24 origin.x			*0.52 origin.x*		
τ_{11}				0.01 origin.x.ctemperature			*0.02 origin.x.ctemperature*		
							0.05 origin.x.I(ctemperature^2)		
ρ_{01}				-1.00 origin.x			*1.00 origin.x.ctemperature*		
							-1.00 origin.x.I(ctemperature^2)		
ICC	0.87								
N	14 ctemperature			14 ctemperature			*14 ctemperature*		
	5 origin.x			5 origin.x			*5 origin.x*		
Observations	70			70			*70*		
Marginal R^2 / Conditional R^2	0.298 / 0.906			0.298 / 0.909			*0.297 / 0.926*		
AIC	190.234			192.716			*188.88*		
log-Likelihood	-89.134			-88.381			*-83.493*		

Table S6. Summary of random regression mixed models (RRMMs) with PC2 on metabolites as response variable and cumulated precipitation as fixed variables. Values in bold indicate a significant effect at *P* < 0.05. The following models were tested: M1. Linear model with random effect (intercept) of (1|receptor), M2. Basic linear model with random effect (intercept) of (1|receptor) and (1|origin), M3. Basic linear model with random effect of origin vary both in intercept and slope (1|receptor)+ (1+receptor|orign), M4.Quadric fixed-effect model (quadric polynomial) with random effect (intercept) of (1|receptor), M5. Quadric fixed-effect model (quadric polynomial) with random effects (intercept) of (1|receptor) and (1|origin), M6. Quadric fixed-effect model (quadric polynomial) with random effect of origin vary both in intercept and slope (1|receptor)+ (1+receptor|orign), M7. Quadric fixed-effect model (quadric polynomial) with random effect of origin vary both in intercept and slope and curvature (1|receptor)+ (1+receptor^2|orign). The best model is highlighted italicized.

All seasons												
	M1			M2			M3			M4		
Predictors	Estimates	Statistic	p	Estimates	Statistic	p	Estimates	Statistic	p	Estimates	Statistic	p
(Intercept)	-0.03	-0.16	0.870	-0.03	-0.12	0.901	-0.03	-0.12	0.902	-0.29	-1.14	0.26
ctemperature	-0.87	-4.07	**<0.001**	-0.87	-4.01	**<0.001**	-0.87	-3.75	**<0.001**			
ctemperature, 2 [1st degree]										-1.32	-3.81	**<0.001**
ctemperature, 2 [2nd degree]										0.26	1.57	0.121
**Random Effects**												
σ^2	0.42			0.25			0.21			0.42		
τ_{00}	0.55 ctemperature			0.60 ctemperature<br>0.16 origin.x			0.61 ctemperature<br>0.16 origin.x			0.45 ctemperature		
τ_{11}							0.03 origin.x.ctemperature					
ρ_{01}							-0.51 origin.x					
ICC	0.56			0.76			0.79			0.52		
N	14 ctemperature			14 ctemperature<br>5 origin.x			14 ctemperature<br>5 origin.x			14 ctemperature		
Observations	70			70			70			70		
Marginal R^2 / Conditional R^2	0.438 / 0.755			0.430 / 0.861			0.427 / 0.882			0.494 / 0.755		
AIC	177.261			158.329			157.873			179.06		

log-Likelihood	-83.393	-73.216	-71.054	-82.255

	M5			M6			M7		
Predictors	Estimates	Statistic	p	Estimates	Statistic	p	Estimates	Statistic	p
(Intercept)	-0.29	-0.92	0.361	*-0.29*	*-0.92*	*0.363*	-0.29	-0.96	0.343
ctemperature ctemperature, 2 [1st degree]	-1.32	-3.75	**<0.001**	*-1.32*	*-3.64*	***0.001***	-1.32	-3.54	**0.001**
ctemperature, 2 [2nd degree]	0.26	1.55	0.127	*0.26*	*1.54*	*0.128*	0.26	1.52	0.134
**Random Effects**									
σ^2	0.25			*0.21*			0.2		
τ_{00}	0.51 ctemperature<br>0.16 origin.x			*0.52 ctemperature*<br>*0.16 origin.x*			0.52 ctemperature<br>0.12 origin.x		
τ_{11}				*0.03 origin.x.ctemperature*			0.07 origin.x.ctemperature<br>0.00 origin.x.I(ctemperature^2)		
ρ_{01}				*-0.51 origin.x*			-0.69 origin.x.ctemperature<br>0.95 origin.x.I(ctemperature^2)		
ICC	0.73			*0.76*					
N	14 ctemperature<br>5 origin.x			*14 ctemperature*<br>*5 origin.x*			14 ctemperature<br>5 origin.x		
Observations	70			*70*			70		
Marginal R^2 / Conditional R^2	0.485 / 0.860			*0.491 / 0.880*			0.480 / 0.890		
AIC	160.097			*159.632*			164.118		
log-Likelihood	-72.113			*-69.959*			-69.212		

Table S7. Summary of linear effect mixed models (LME) testing the effect of receptor site, species and season (fixed effects) on *Sphagnum* water content, WEOM, WEOC, GEP and *Sphagnum* metabolites with plot nested into block and receptor site as a random effect on the intercept. Values in bold indicate significant effects at $P < 0.05$.

	**species**		**receptor**		**species*receptor**		**season**		**season*species**		**season*receptor**		**receptor*season*species**	
	F	*P-value*	*F*	*P-value*	*F*	*P-value*	*F*	*P-value*	*F*	*P-value*	*F*	*P-value*	*F*	*P-value*
**All seasons**														
	NumDF=4	DenDF=230	NumDF=4	DenDF=231	NumDF=16	DenDF=229	NumDF=2	DenDF=9.4	NumDF=8	DenDF=230	NumDF=7	DenDF=231	NumDF=27	DenDF=230
Sphagnum water content	5.185	***0.001***	1.293	*0.278*	1.037	*0.423*	166.301	***0.0001***	1.090	*0.371*	18.152	***0.0001***	0.571	*0.957*
WEOC	12.949	***0.0001***	1.317	*1.000*	1.286	*0.207*	162.995	***0.0001***	3.700	***0.0001***	10.016	***0.0001***	0.581	*0.953*
WEON	2.212	*0.070*	0.588	*1.000*	0.683	*0.807*	11.949	***0.0001***	0.660	*0.727*	13.010	***0.0001***	0.755	*0.804*
	NumDF=4	DenDF=208	NumDF=4	DenDF=210	NumDF=16	DenDF=206	NumDF=2	DenDF=210	NumDF=8	DenDF=207	NumDF=7	DenDF=208	NumDF=27	DenDF=206
GEP	8.973	***0.000***	1.355	*0.252*	1.865	***0.028***	39.254	***0.0001***	4.424	***0.0001***	8.846	***0.0001***	1.209	*0.230*
NEE	12.476	***<0.001***	18.685	***<0.001***	1.141	*0.3175*	18.235	***<0.001***	8.689	***<0.001***	10.3	***<0.001***	0.671	*0.896*
ER	30.695	***<0.001***	2.339	*0.055*	2.504	***<0.001***	176.22	***<0.001***	6.469	***<0.001***	3.349	***<0.001***	1.312	*0.142*
	NumDF=4	DenDF=261	NumDF=4	DenDF=261	NumDF=16	DenDF=261	NumDF=2	DenDF=261	NumDF=8	DenDF=261	NumDF=7	DenDF=261	NumDF=28	DenDF=261
water-soluble phenols	38.297	***0.000***	6.813	***0.000***	2.059	***0.013***	338.236	***0.000***	5.590	***0.000***	16.072	***0.000***	1.039	*0.418*
chlorophyll a	8.321	***0.000***	1.960	*1.000*	2.533	***0.002***	462.012	***0.000***	1.793	*0.079*	21.563	***0.000***	2.790	***0.000***
chlorophyll b	26.019	***0.000***	1.501	*0.204*	1.978	***0.018***	324.202	***0.000***	5.906	***0.000***	33.330	***0.000***	1.755	***0.014***
carotenoids	13.357	***0.000***	0.892	*0.470*	2.687	***0.001***	182.400	***0.000***	1.916	*0.059*	18.337	***0.000***	2.269	***0.001***
proline	9.935	***0.000***	1.845	*0.122*	2.210	***0.007***	379.628	***0.000***	1.518	*0.152*	24.563	***0.000***	2.500	***0.000***
total carbohydrates	18.845	***0.000***	0.933	*0.446*	3.301	***0.000***	71.749	***0.000***	1.331	*0.229*	8.244	***0.000***	3.179	***0.000***

total tannins	23.355	***0.000***	0.782	0.538	1.552	0.082	18.516	***0.000***	5.472	***0.000***	16.276	***0.000***	2.093	***0.002***
total phenols	11.351	***0.000***	1.865	0.119	2.334	***0.003***	122.582	***0.000***	3.234	***0.002***	29.195	***0.000***	2.582	***0.000***
total flavonoids	27.488	***0.000***	1.526	0.197	1.507	0.106	293.111	***0.000***	7.268	***0.000***	29.360	***0.000***	2.110	***0.002***
PC1 metabolites	10.888	***0.000***	2.455	***0.047***	2.941	***0.000***	201.978	***0.000***	3.675	***0.000***	14.377	***0.000***	2.158	***0.001***
PC2 metabolites	24.537	***0.000***	2.008	0.095	1.267	0.226	529.481	***0.000***	5.373	***0.000***	20.898	***0.000***	2.254	***0.001***

Spring

	NumDF=4	DenDF=95	NumDF=4	DenDF=95	NumDF=16	DenDF=95
Sphagnum water content	6.615	***0.000***	2.926	***0.026***	1.284	0.236
WEOC	2.775	***0.036***	27.441	***0.000***	1.166	0.317
WEON	0.621	0.649	14.397	***0.000***	0.710	0.775
	NumDF=4	DenDF=91.1	NumDF=4	DenDF=91.1	NumDF=16	DenDF=91
GEP	30.997	***<0.001***	13.759	***0.000***	4.098	***0.000***
NEE	415.08	***<0.001***	24.606	***<0.001***	2.006	***0.021***
ER	265.94	***<0.001***	10.48	***<0.001***	3.701	***<0.001***
	NumDF=4	DenDF=96	NumDF=4	DenDF=96	NumDF=16	DenDF=96
water-soluble phenols	11.112	***0.000***	20.378	***0.000***	1.132	0.342
chlorophyll a	5.286	***0.001***	12.694	***0.000***	6.106	***0.000***
chlorophyll b	2.390	0.062	8.812	***0.000***	5.176	***0.000***
carotenoids	5.288	***0.001***	14.519	***0.000***	5.228	***0.000***
proline	8.676	***0.000***	9.009	***0.000***	2.319	***0.006***
total carbohydrates	25.507	***0.000***	95.821	***0.000***	3.062	***0.000***

total tannins	6.718	***0.000***	35.311	***0.000***	2.596	***0.002***
total phenols	8.541	***0.000***	42.601	***0.000***	2.842	***0.002***
total flavonoids	8.277	***0.000***	18.962	***0.000***	2.250	***0.008***
PC1 metabolites	7.711	***0.000***	50.254	***0.000***	4.285	***0.000***
PC2 metabolites	13.644	***0.000***	19.419	***0.000***	2.618	***0.003***
**Summer**						
	NumDF=4	DenDF=72	NumDF=4	DenDF=73	NumDF=16	DenDF=72
Sphagnum water content	0.959	0.437	16.370	***0.0001***	0.520	0.928
WEOC	6.030	***0.0001***	10.608	***0.0001***	0.475	0.952
WEON	1.792	0.139	11.209	***0.0001***	0.424	0.972
	NumDF=4	DenDF=71	NumDF=4	DenDF=72	NumDF=16	DenDF=71
GEP	6.415	***0.0001***	5.353	***0.001***	1.082	0.388
NEE	18.19	***<0.001***	11.572	***<0.001***	1.144	0.33
ER	22.78	***<0.001***	1.80	0.136	1.06	0.403
	NumDF=4	DenDF=92	NumDF=4	DenDF=92	NumDF=16	DenDF=92
water-soluble phenols	32.545	***0.000***	26.070	***0.000***	1.717	0.057
chlorophyll a	3.949	***0.005***	43.376	***0.000***	1.361	0.180
chlorophyll b	7.917	***0.000***	30.222	***0.000***	0.910	0.560
carotenoids	4.832	***0.001***	39.997	***0.000***	1.261	0.241
proline	6.287	***0.000***	18.529	***0.000***	3.595	***0.000***
total carbohydrates	16.587	***0.000***	2.901	***0.026***	1.728	0.055
total tannins	7.484	***0.000***	15.804	***0.000***	2.493	***0.003***
total phenols	27.621	***0.000***	16.621	***0.000***	1.587	0.088
total flavonoids	9.754	***0.000***	44.892	***0.000***	2.948	***0.001***

PC1 metabolites	20.640	***0.000***	37.687	***0.000***	0.838	0.640
PC2 metabolites	14.092	***0.000***	38.347	***0.000***	1.304	0.213
**Autumn**						
	NumDF=4	DenDF=44	NumDF=3	DenDF=44	NumDF=11	DenDF=43
Sphagnum water content	2.614	***0.043***	5.556	***0.002***	0.586	0.833
WEOC	1.590	0.188	2.947	***0.040***	1.299	0.247
WEON	0.926	0.454	1.330	0.273	1.379	0.206
	NumDF=4	DenDF=45	NumDF=4	DenDF=45	NumDF=16	DenDF=44
GEP	2.570	0.051	11.294	***0.000***	0.679	0.750
NEE	1.804	0.1430	9.73	***<0.001***	0.317	0.963
ER	2.648	***0.044***	0.365	0.778	1.149	0.344
	NumDF=4	DenDF=73	NumDF=3	DenDF=73	NumDF=12	DenDF=73
water-soluble phenols	15.471	***0.000***	5.547	***0.002***	1.904	0.052
chlorophyll a	2.979	***0.033***	43.428	***0.000***	2.277	***0.018***
chlorophyll b	4.124	***0.009***	47.306	***0.000***	1.334	0.224
carotenoids	2.798	***0.041***	47.082	***0.000***	2.211	***0.022***
proline	4.870	***0.004***	1.886	0.143	3.510	***0.001***
total carbohydrates	3.095	***0.028***	9.494	***0.000***	1.724	0.083
total tannins	1.154	0.339	16.328	***0.000***	1.173	0.320
total phenols	5.151	***0.003***	17.074	***0.000***	1.406	0.189
total flavonoids	2.095	0.110	29.197	***0.000***	2.238	***0.021***
PC1 metabolites	2.155	0.095	63.276	***0.000***	2.055	***0.034***
PC2 metabolites	6.801	***0.001***	16.005	***0.000***	1.341	0.220

Table S8. Summary of linear effect mixed models (LME) testing the effect of mean temperature and precipitation (fixed effects) on *Sphagnum* water content, WEOM, WEOC, gross ecosystem productivity and *Sphagnum* metabolites with plot nested into block and receptor site as a random effect on the intercept. Values in bold indicate significant effects at $P < 0.05$.

	**temperature**		**precipitation**		**temperature*precipitation**	
	F	*P-value*	*F*	*P-value*	*F*	*P-value*
**All seasons**						
	NumDF=1	DenDF=281	NumDF=1	DenDF=263	NumDF=1	DenDF=282
Sphagnum water content	19.51261	***<0.001***	0.258588	0.6118	2.076289767	0.151637
WEOC	110.7986	***<0.001***	19.05631	***<0.001***	58.79034963	***<0.001***
WEON	53.65297	***<0.001***	22.47428	***<0.001***	39.32660814	***<0.001***
	NumDF=1	DenDF=281	NumDF=1	DenDF=224	NumDF=1	DenDF=279
GEP	0.7039	0.4029	2.5041	0.1165	0.6718	0.4141
NEE	1.762	0.185	3.536	0.061	9.540	***0.0022***
ER	135.61	***<0.001***	12.59	***<0.001***	18.21	***<0.001***
	NumDF=1	DenDF=281	NumDF=1	DenDF=263	NumDF=1	DenDF=282
water-soluble phenols	22.11212	***<0.001***	0.053016	0.819	3.305287529	0.076169
chlorophyll a	33.84334	***<0.001***	32.23767	***<0.001***	0.221596698	0.638187
chlorophyll b	31.30955	***<0.001***	26.63082	***<0.001***	2.178664959	0.141486
carotenoids	33.61474	***<0.001***	30.68959	***<0.001***	0.420104324	0.517411
proline	2.707525	0.103252	0.204915	0.6523	1.655067948	0.203079
total carbohydrates	24.18402	***<0.001***	13.92566	***<0.001***	14.52627285	***<0.001***
total tannins	2.696277	0.101619	22.86038	***<0.001***	15.30688751	***<0.001***
total phenols	35.48832	***<0.001***	5.831441	***<0.001***	5.99097319	***<0.001***
total flavonoids	33.50533	***<0.001***	7.71158	***<0.001***	0.466464736	0.495425
PC1 metabolites	65.22791	***<0.001***	25.19277	***<0.001***	1.308878343	0.253896
PC2 metabolites	0.016212	0.898824	11.06813	***<0.001***	0.003363738	0.953834
**Spring**						
	NumDF=1	DenDF=116	NumDF=1	DenDF=116	NumDF=1	DenDF=116
Sphagnum water content	3.09502	0.081902	1.853064	0.1768	0.408196598	0.524513
WEOC	5.711912	***0.018841***	2.685409	0.1046	30.53870348	***<0.001***
WEON	50.92885	***<0.001***	50.61291	***<0.001***	57.61239621	***<0.001***
	NumDF=1	DenDF=112	NumDF=1	DenDF=112	NumDF=1	DenDF=112

GEP	20.909	***<0.001***	0.942	0.334	6.515	0.012
NEE	13.645	***0.0004***	1.217	0.272	16.64	***<0.001***
ER	12.322	***0.0007***	3.907	*0.051*	0.613	0.436
	NumDF=1	DenDF=117	NumDF=1	DenDF=117	NumDF=1	DenDF=117
water-soluble phenols	20.37849	***<0.001***	11.11225	***<0.001***	1.132208938	0.341726
chlorophyll a	12.69372	***<0.001***	5.286133	***<0.001***	6.105525698	***<0.001***
chlorophyll b	8.811885	***<0.001***	2.389896	0.0617	5.175999255	***<0.001***
carotenoids	14.51852	***<0.001***	5.288385	***<0.001***	5.227610168	***<0.001***
proline	9.008504	***<0.001***	8.675791	***<0.001***	2.319389223	***<0.001***
total carbohydrates	95.82088	***<0.001***	25.5072	***<0.001***	3.06206108	***<0.001***
total tannins	35.31063	***<0.001***	6.717501	***<0.001***	2.596177189	***<0.001***
total phenols	42.6011	***<0.001***	8.540502	***<0.001***	2.84199307	***<0.001***
total flavonoids	18.96183	***<0.001***	8.276506	***<0.001***	2.249752018	***<0.001***
PC1 metabolites	50.2537	***<0.001***	7.711271	***<0.001***	4.284944186	***<0.001***
PC2 metabolites	19.41872	***<0.001***	13.64444	***<0.001***	2.617938578	***<0.001***
**Summer**						
	NumDF=1	DenDF=96	NumDF=1	DenDF=96	NumDF=1	DenDF=96
Sphagnum water content	35.7930	***0.0000***	43.1900	***0.0000***	40.3840	***0.0000***
WEOC	0.0008	0.9771	0.8631	0.3551	0.2545	0.6150
WEON	12.7312	***0.0006***	7.0195	***0.0094***	10.8363	***0.0014***
	NumDF=1	DenDF=96	NumDF=1	DenDF=96	NumDF=1	DenDF=96
GEP	2.383	0.125	6.014	***0.016***	5.258	***0.024***
NEE	0.0005	0.994	0.366	0.546	13.746	***0.0004***
ER	0.268	0.605	1.288	0.259	1.718	0.193
	NumDF=1	DenDF=113	NumDF=1	DenDF=113	NumDF=1	DenDF=113
water-soluble phenols	26.06995	***<0.001***	32.54489	***<0.001***	1.717112292	0.056879
chlorophyll a	43.37638	***<0.001***	3.949485	***0.0054***	1.361116046	0.180219
chlorophyll b	30.22167	***<0.001***	7.917212	***<0.001***	0.910365031	0.559867
carotenoids	39.99716	***<0.001***	4.832022	***0.0014***	1.260705755	0.240698
proline	18.52875	***<0.001***	6.286515	***0.0002***	3.595069591	***<0.001***
total carbohydrates	2.90092	***0.026062***	16.58715	***<0.001***	1.727828769	0.05483

total tannins	15.80357	***<0.001***	7.483649	***<0.001***	2.493456229	***0.003342***
total phenols	16.62051	***<0.001***	27.62097	***<0.001***	1.58735793	0.08791
total flavonoids	44.89159	***<0.001***	9.754077	***<0.001***	2.948014578	***0.00063***
PC1 metabolites	37.68683	***<0.001***	20.64048	***<0.001***	0.83782015	0.640191
PC2 metabolites	38.34698	***<0.001***	14.09175	***<0.001***	1.304038755	0.212802
			**Autumn**			
	NumDF=1	DenDF=59	NumDF=1	DenDF=60	NumDF=1	DenDF=62
Sphagnum water content	13.1466	***0.0005***	11.6320	***0.0010***	15.6176	***0.0002***
WEOC	1.8309	0.1800	1.0596	0.3066	1.8211	0.1812
WEON	0.0277	0.8682	0.0518	0.8206	0.6833	0.4110
	NumDF=1	DenDF=62	NumDF=1	DenDF=62	NumDF=1	DenDF=62
GEP	32.845	***<0.001***	35.01	***<0.001***	40.918	***<0.001***
NEE	0.695	0.41	5.074	***0.029***	24.99	***<0.001***
ER	0.014	0.904	0.110	0.741	0.452	0.505
	NumDF=1	DenDF=89	NumDF=1	DenDF=89	NumDF=1	DenDF=89
water-soluble phenols	5.546867	***0.002129***	15.47087	***<0.001***	1.904475302	0.051702
chlorophyll a	43.42828	***<0.001***	2.97936	***0.0326***	2.277077729	***0.018046***
chlorophyll b	47.30605	***<0.001***	4.123598	***0.0091***	1.334229233	0.223687
carotenoids	47.08185	***<0.001***	2.797935	***0.0412***	2.211399627	***0.021784***
proline	1.885617	0.142937	4.869611	***0.0037***	3.510209162	***0.000616***
total carbohydrates	9.493672	***<0.001***	3.095342	***0.028***	1.723907995	0.082996
total tannins	16.32774	***<0.001***	1.153767	0.3388	1.172801366	0.319672
total phenols	17.07359	***<0.001***	5.150551	***0.0028***	1.405762888	0.188701
total flavonoids	29.19742	***<0.001***	2.094621	0.1101	2.23757278	***0.020901***
PC1 metabolites	63.27561	***<0.001***	2.154506	0.0955	2.054935189	***0.033951***
PC2 metabolites	16.00485	***<0.001***	6.800997	***0.0005***	1.341458151	0.21952

Table S9. Summary of linear effect mixed models (*lmer*) testing the effect of delta temperature and delta precipitation (fixed effects) on *Sphagnum* water content, WEOM, WEOC, gross ecosystem productivity and *Sphagnum* metabolites with plot nested into block and receptor site as a random effect on the intercept. Values in bold indicate significant effects at $P < 0.05$.

	**Δ temperature**		**Δ precipitation**		**Δ temperature*precipitation**	
	F	*P-value*	*F*	*P-value*	*F*	*P-value*
**All seasons**						
	NumDF=1	DenDF=281	NumDF=1	DenDF=263	NumDF=1	DenDF=282
Sphagnum water content	75.82903	***<0.001***	22.03818	***<0.001***	0.9665368	0.3267378
WEOC	61.50631	***<0.001***	10.17109	***0.001587***	0.8624309	0.3538479
WEON	9.64266	***0.002514***	2.575915	0.110261	0.0600281	0.8066633
	NumDF=1	DenDF=281	NumDF=1	DenDF=224	NumDF=1	DenDF=279
GEP	3.7477	*0.054*	0.0538	0.8167	0.1750	0.6760
NEE	1.021	0.313	8.346	***0.004***	0.265	0.607
ER	22.58	***<0.001***	2.763	0.097	13.972	***0.002***
	NumDF=1	DenDF=281	NumDF=1	DenDF=263	NumDF=1	DenDF=282
water-soluble phenols	62.42958	***<0.001***	6.519341	***0.011218***	0.0179495	0.8935105
chlorophyll a	25.49454	***<0.001***	120.2933	***<0.001***	1.3440187	0.2472362
chlorophyll b	39.4207	***<0.001***	92.65202	***<0.001***	0.4876159	0.4857467
carotenoids	33.72875	***<0.001***	117.6815	***<0.001***	0.5161435	0.4730451
proline	18.7843	***<0.001***	1.867312	0.173167	6.8534948	***0.0093538***
total carbohydrates	8.169528	***0.004647***	4.791684	***0.029401***	0.0478383	0.8270115
total tannins	1.787677	0.18637	1.69408	0.195522	13.751638	***0.0002727***
total phenols	14.03781	***0.000391***	8.268145	***0.004725***	1.473388	0.2262473
total flavonoids	69.38749	***<0.001***	42.88659	***<0.001***	7.3145666	***0.0072413***
PC1 metabolites	81.15754	***<0.001***	104.5304	***<0.001***	1.1553417	0.283244
PC2 metabolites	7.348963	***<0.001***	8.617931	***0.003658***	0.3730803	0.541819
**Spring**						
	NumDF=1	DenDF=116	NumDF=1	DenDF=116	NumDF=1	DenDF=116
Sphagnum water content	2.690533	0.104416	2.295664	0.133168	0.4081966	0.5245131
WEOC	60.8152	***<0.001***	0.226239	0.635413	30.538703	***<0.001***
WEON	16.92476	***<0.001***	45.1228	***<0.001***	57.612334	***<0.001***
	NumDF=1	DenDF=112	NumDF=1	DenDF=112	NumDF=1	DenDF=112
GEP	4.848	***0.029***	2.061	0.153	6.515	***0.0123***

NEE	13.648	***0.0004***	1.217	0.272	16.646	***0.0001***
ER	12.322	***0.0007***	3.907	*0.051*	0.612	0.435
	NumDF=1	DenDF=117	NumDF=1	DenDF=117	NumDF=1	DenDF=117
water-soluble phenols	1.196949	0.27618	21.43864	***<0.001***	9.680371	***0.0023405***
chlorophyll a	16.4788	***0.000101***	9.149769	***0.003189***	19.369425	***<0.001***
chlorophyll b	19.91638	***<0.001***	0.30696	0.580843	5.88817	***0.0171554***
carotenoids	28.84555	***<0.001***	4.687184	***0.032866***	19.701667	***<0.001***
proline	0.303195	0.583218	0.011536	0.914697	1.8220831	0.1803977
total carbohydrates	72.30027	***<0.001***	264.0742	***<0.001***	263.22563	***<0.001***
total tannins	23.55282	***<0.001***	69.89641	***<0.001***	77.152731	***<0.001***
total phenols	0.01287	0.909921	37.14455	***<0.001***	15.290605	***<0.001***
total flavonoids	31.60474	***<0.001***	25.79134	***<0.001***	38.570561	***<0.001***
PC1 metabolites	29.86893	***<0.001***	44.71843	***<0.001***	51.584371	***<0.001***
PC2 metabolites	1.572256	0.213018	25.26612	***<0.001***	4.8622087	***0.0299388***
			**Summer**			
	NumDF=1	DenDF=96	NumDF=1	DenDF=96	NumDF=1	DenDF=96
Sphagnum water content	35.56169	***<0.001***	42.16629	***<0.001***	39.744231	***<0.001***
WEOC	0.20927	0.648333	3.383274	0.068829	1.4403009	0.2329281
WEON	16.60014	***<0.001***	9.292992	***0.002946***	14.385646	***0.0002557***
	NumDF=1	DenDF=96	NumDF=1	DenDF=96	NumDF=1	DenDF=96
GEP	0.304	0.582	6.377	***0.0135***	5.258	***0.0240***
NEE	0.0005	0.994	0.366	0.546	13.746	***0.0004***
ER	0.268	0.605	1.288	0.193	1.718	0.193
	NumDF=1	DenDF=113	NumDF=1	DenDF=113	NumDF=1	DenDF=113
water-soluble phenols	1.889285	0.172708	10.55107	***0.001619***	18.629119	***<0.001***
chlorophyll a	28.50323	***<0.001***	14.62959	***0.000218***	0.0215054	0.8836813
chlorophyll b	33.6378	***<0.001***	5.667566	***0.018955***	0.3091916	0.579276
carotenoids	31.46027	***<0.001***	12.44824	***0.000614***	0.009451	0.922733
proline	22.96822	***<0.001***	1.959031	0.164356	8.4382247	***0.0044209***
total carbohydrates	2.932585	0.090281	5.424716	***0.022059***	0.3512421	0.5548828
total tannins	34.55001	***<0.001***	0.922088	0.338978	19.431405	***2.387E-05***
total phenols	4.035945	***0.047538***	2.949588	0.089263	0.7938571	0.3752721
total flavonoids	65.19689	***<0.001***	20.93833	***<0.001***	23.880788	***<0.001***

PC1 metabolites	10.60988	***0.001486***	4.147616	***0.044031***	1.3284157	0.2515197
PC2 metabolites	80.79255	***<0.001***	3.6493	0.059165	24.48911	***<0.001***
			**Autumn**			
	NumDF=1	DenDF=59	NumDF=1	DenDF=60	NumDF=1	DenDF=62
Sphagnum water content	9.2793	***0.0031***	0.2157	0.6436	15.6176	***0.0002***
WEOC	1.4500	0.2322	0.0105	0.9186	1.8211	0.1812
WEON	0.4637	0.4980	2.9198	0.0916	0.6833	0.4110
	NumDF=1	DenDF=62	NumDF=1	DenDF=62	NumDF=1	DenDF=62
GEP	21.32	***<0.001***	0.266	0.608	40.91	***<0.001***
NEE	0.695	0.408	5.073	***0.029***	24.99	***<0.001***
ER	0.014	0.904	0.110	0.741	0.451	0.505
	NumDF=1	DenDF=89	NumDF=1	DenDF=89	NumDF=1	DenDF=89
water-soluble phenols	0.004082	0.949203	2.65465	0.106782	3.6123812	0.0605877
chlorophyll a	57.78986	***<0.001***	6.820016	***0.011139***	103.55687	***<0.001***
chlorophyll b	76.33929	***<0.001***	8.101945	***0.005891***	135.99199	***<0.001***
carotenoids	66.55701	***<0.001***	6.809981	***0.011206***	120.02239	***<0.001***
proline	1.122449	0.293158	3.711812	0.058487	0.0571403	0.8117909
total carbohydrates	5.555519	***0.021199***	1.758885	0.189351	18.464928	***<0.001***
total tannins	38.21146	***<0.001***	39.93044	***<0.001***	9.9839026	***0.0021863***
total phenols	11.79302	***0.000905***	0.103493	0.748432	37.19694	***<0.001***
total flavonoids	22.10072	***<0.001***	0.874122	0.353396	74.028298	***<0.001***

Table S10. Summary of linear models testing the correlation between gross ecosystem productivity (fixed effect) and PC1 and PC2 on metabolites. F and P-values based on one-way ANOVA. Values in bold indicate significant effects at $P < 0.05$.

	Df	*F*	*P*
PC1	1, 306	4.39	**0.037**
PC2	1, 306	2.63	0.11

SUPPLEMENTARY FIGURES

Fig. S1

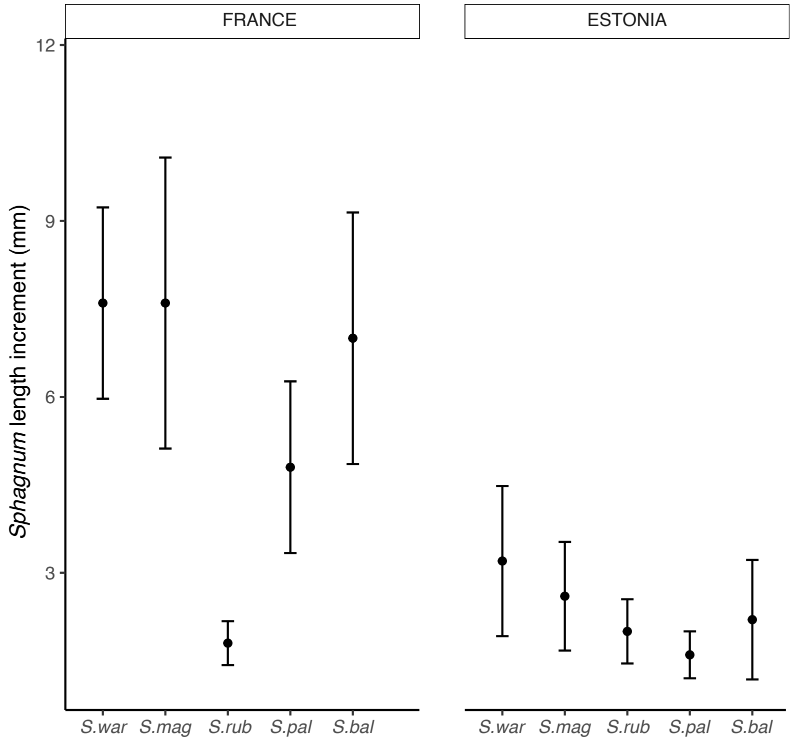

Figure S1. *Sphagnum* length increment in peat-mesocosms from France and Estonia three months after transplantation (from summer to autumn 2018). All *Sphagnum* species show growth in both sites, indicating good acclimation. Data from other sites of the experiment are missing because the cranked wire got rusted and killed *Sphagnum* mosses, preventing growth measurements. n = 5 independent plots per species in each site. *S.war = Sphagnum warnstorfii, S.mag = S. magellanicum, S.rub = S. rubellum, S.pal = S. plaustre, S.bal = S. balticum*.

Fig. S2.

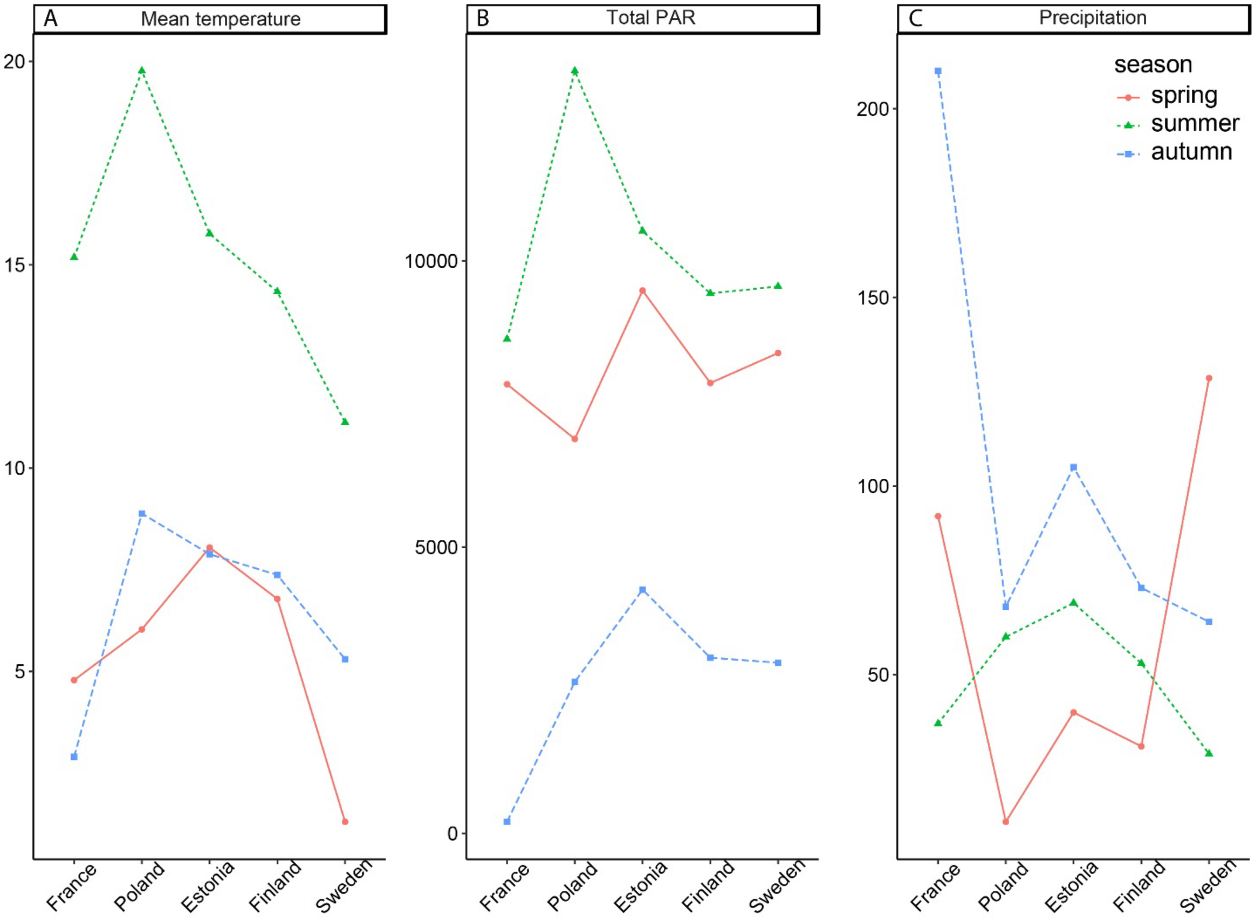

Figure S2. (a) Temperature, (b) total PAR, (c) and precipitation variability in each site for every season. Climatic variables are characterized by mean values of the 30 days period preceding each sampling campaign. Colours of lines correspond to seasons.

Fig. S3

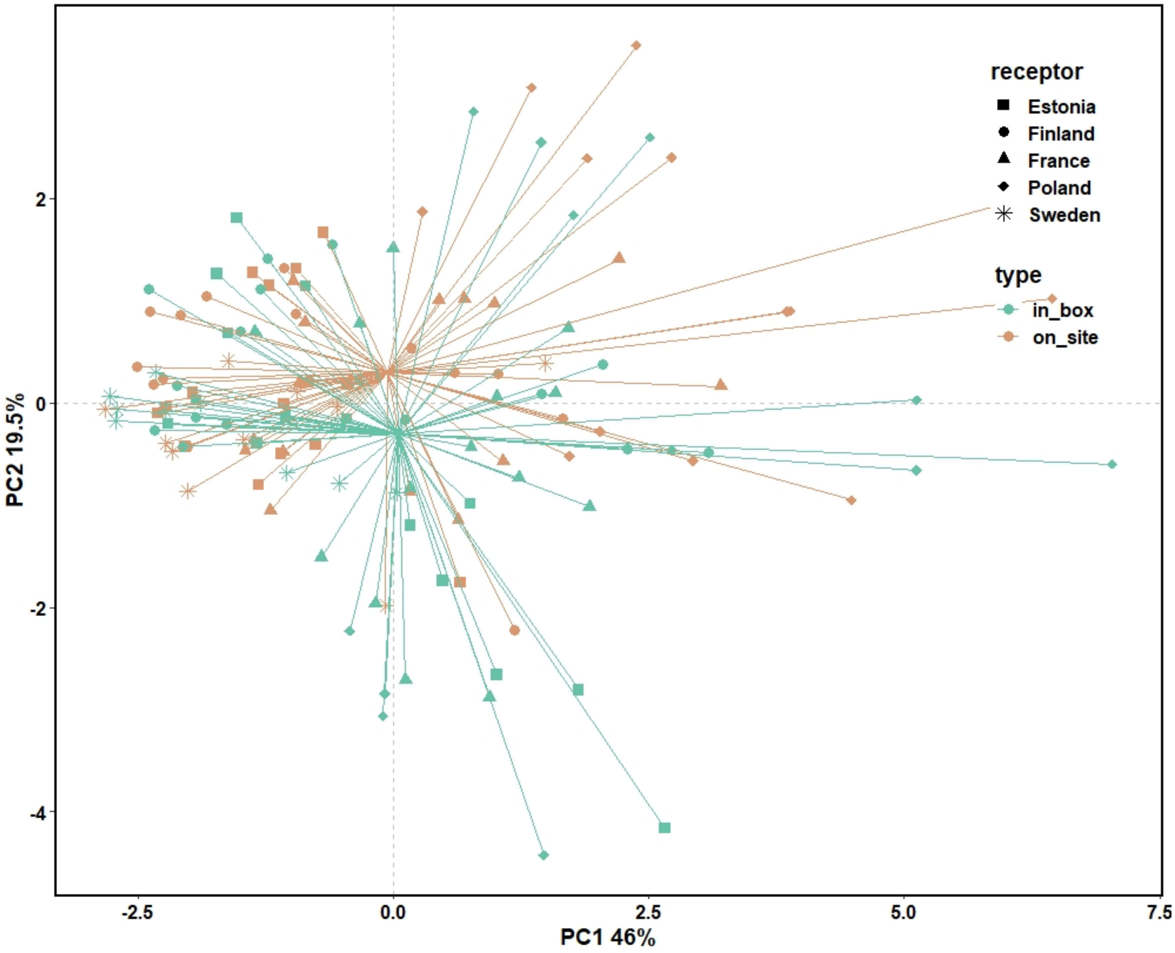

Figure S3. PCA on *Sphagnum* metabolites over the three seasons in the plastic boxes (which stayed at their site of origin) and at the control plots on the sites (untouched plots without boxes). The green color corresponds to the samples collected from the boxes and the orange to the samples in untouched plots. No significant differences between plastic boxes and untouched plots have been found. n = 5 independent plots per condition (with/without plastic box) and site.

Fig. S4

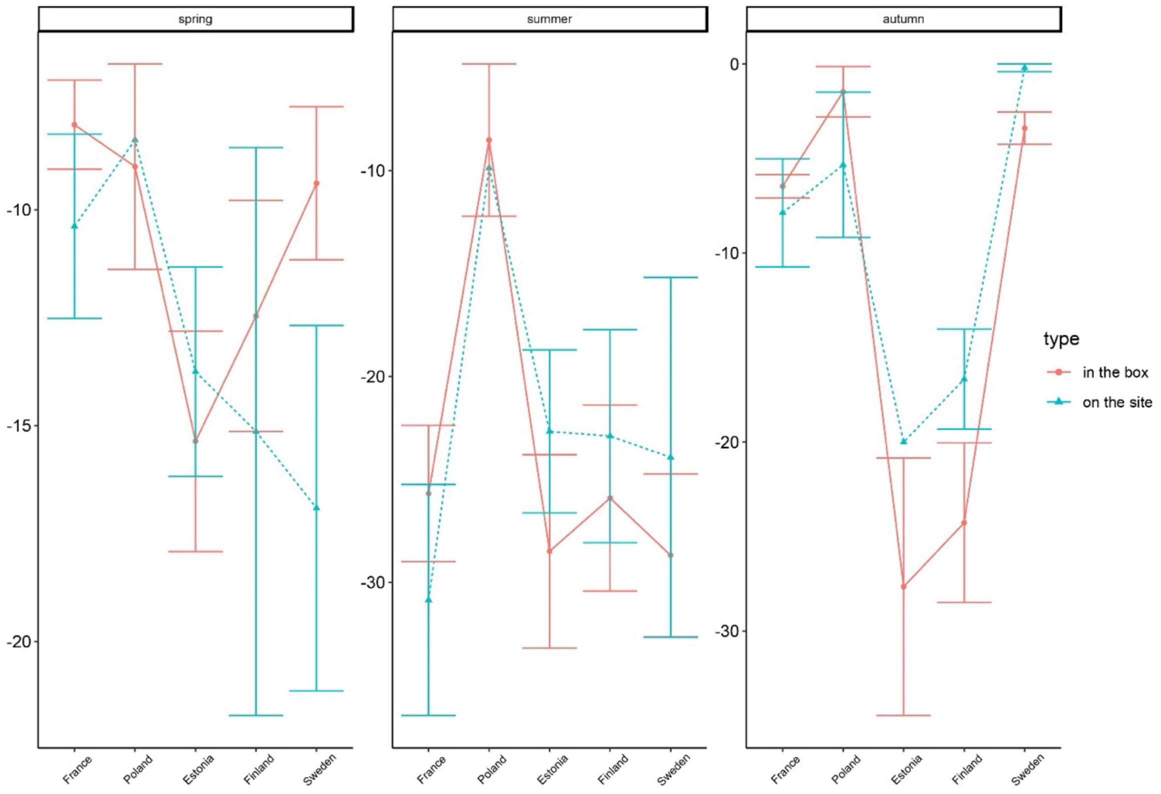

Figure S4. Dynamic of gross ecosystem productivity (GEP) quantified over three seasons in the boxes (which stayed at their site of origin) and at the control plots on the sites (untouched plots without box). Pale red colour corresponds to the samples collected from the boxes and pale blue to samples outside the boxes. No significant effect of plastic boxes has been found. Data are mean ± SE (n = 5 independent plots per condition and per site).

Fig. S5

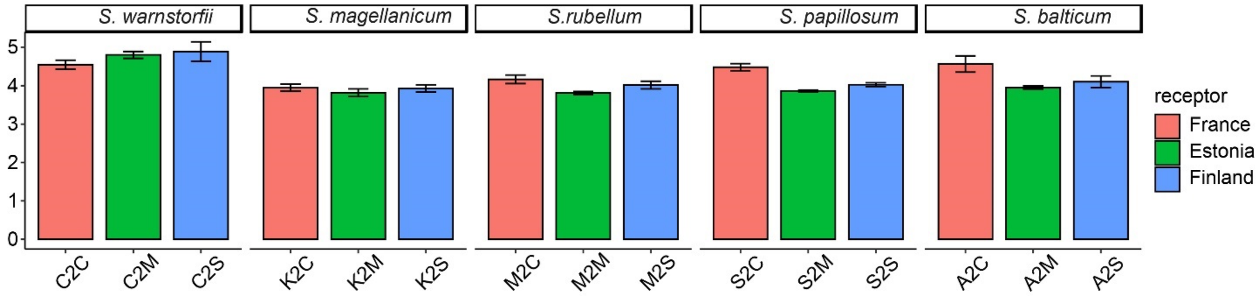

Figure S5. pH values in the plastic boxes from the same site dispatched along the gradient. These measurements have been done only on three sites. Letters indicate the site name, the first letter indicates the donor site and the second letter is the receptor site. C = Coulozouls (France), K = Kusowo (Poland), S = Siikaneva (Finland) and M = Männikjärve (Estonia). No significant transplant effect on pH have been found. Data are mean ± SE (n = 5 independent plots per species and per site).

Fig. S6

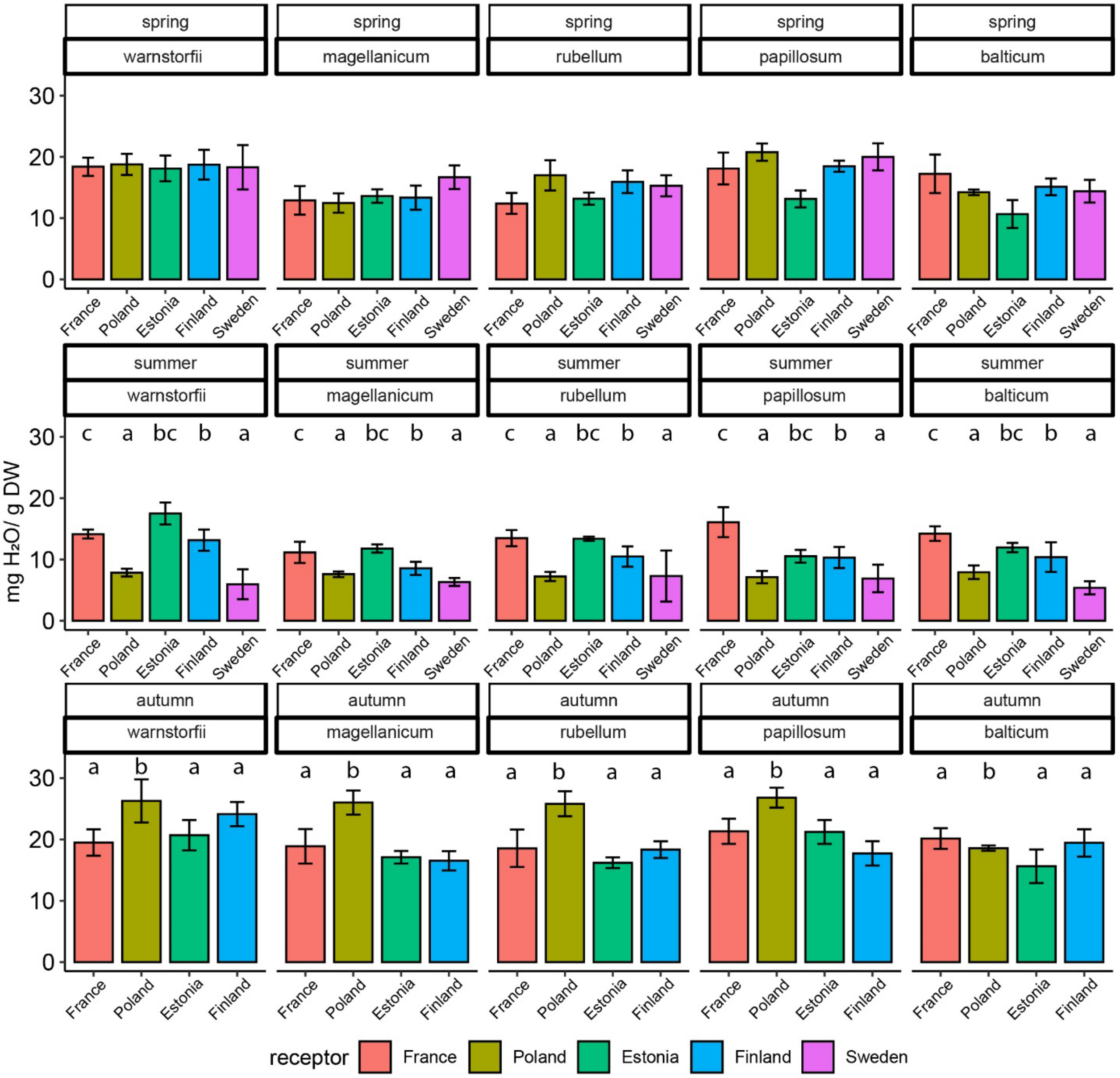

Figure S6. Barplot of *Sphagnum* water content in the transplanted mesocosms for each species across seasons and sites. Data are mean ± standard errors (SE) (n = 5 independent plots per species and per site). Letters indicate significant differences at $P < 0.05$ (LME, post hoc test). warnstorfii: *Sphagnum warnstorfii,* magellanicum: *S. magellanicum*, rubellum: *S. rubellum*, papillosum: *S. papillosum*, balticum: *S. balticum*.

Fig. S7

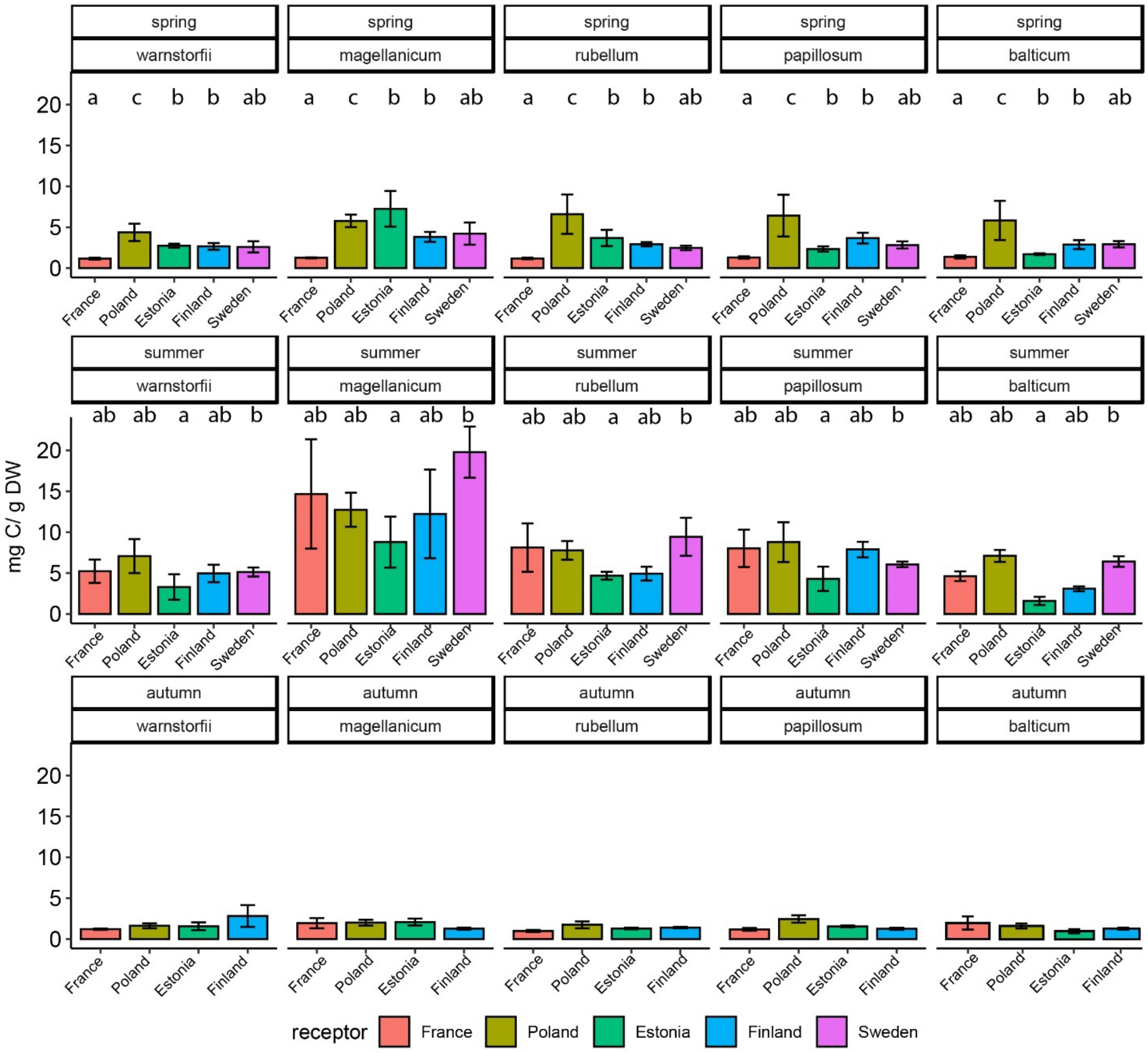

Figure S7. Barplot of water-extractable organic carbon (WEOC) of each *Sphagnum* species collected in the transplanted mesocosms across seasons and sites. Data are mean ± standard errors (SE) (n = 5 independent plots per species and per site). Letters indicate significant differences at $P < 0.05$ (LME, post hoc test). warnstorfii: *Sphagnum warnstorfii*, magellanicum: *S. magellanicum*, rubellum: *S. rubellum*, papillosum: *S. papillosum*, balticum: *S. balticum*.

Fig. S8

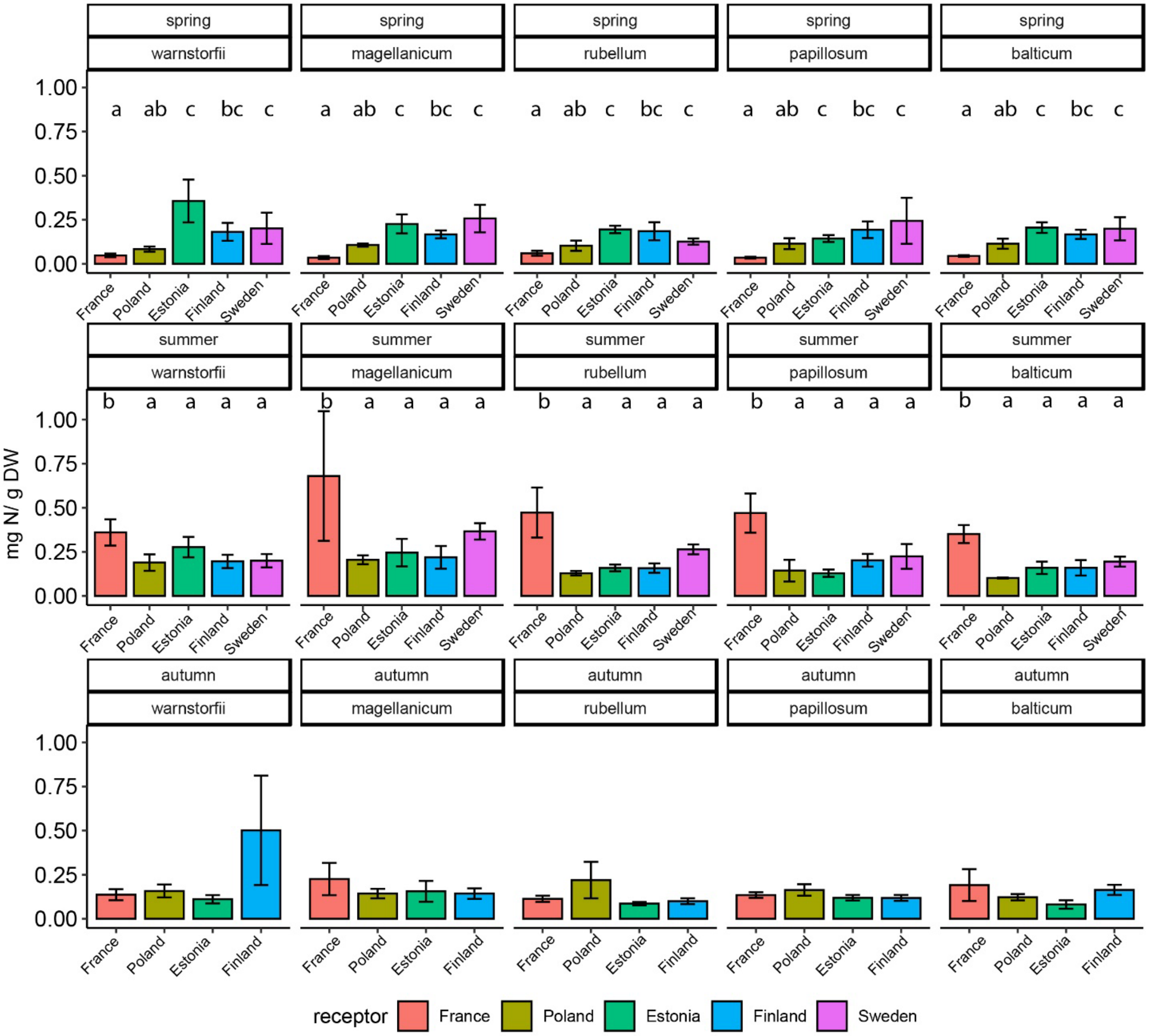

Figure S8. Barplot of water-extractable organic nitrogen (WEON) of each *Sphagnum* species collected in the transplanted mesocosms across seasons and sites. Data are mean ± standard errors (SE) (n = 5 independent plots per species and per site). Letters indicate significant differences at $P < 0.05$ (LME, post hoc test). warnstorfii: *Sphagnum warnstorfii*, magellanicum: *S. magellanicum*, rubellum: *S. rubellum*, papillosum: *S. papillosum*, balticum: *S. balticum*.

Fig. S9

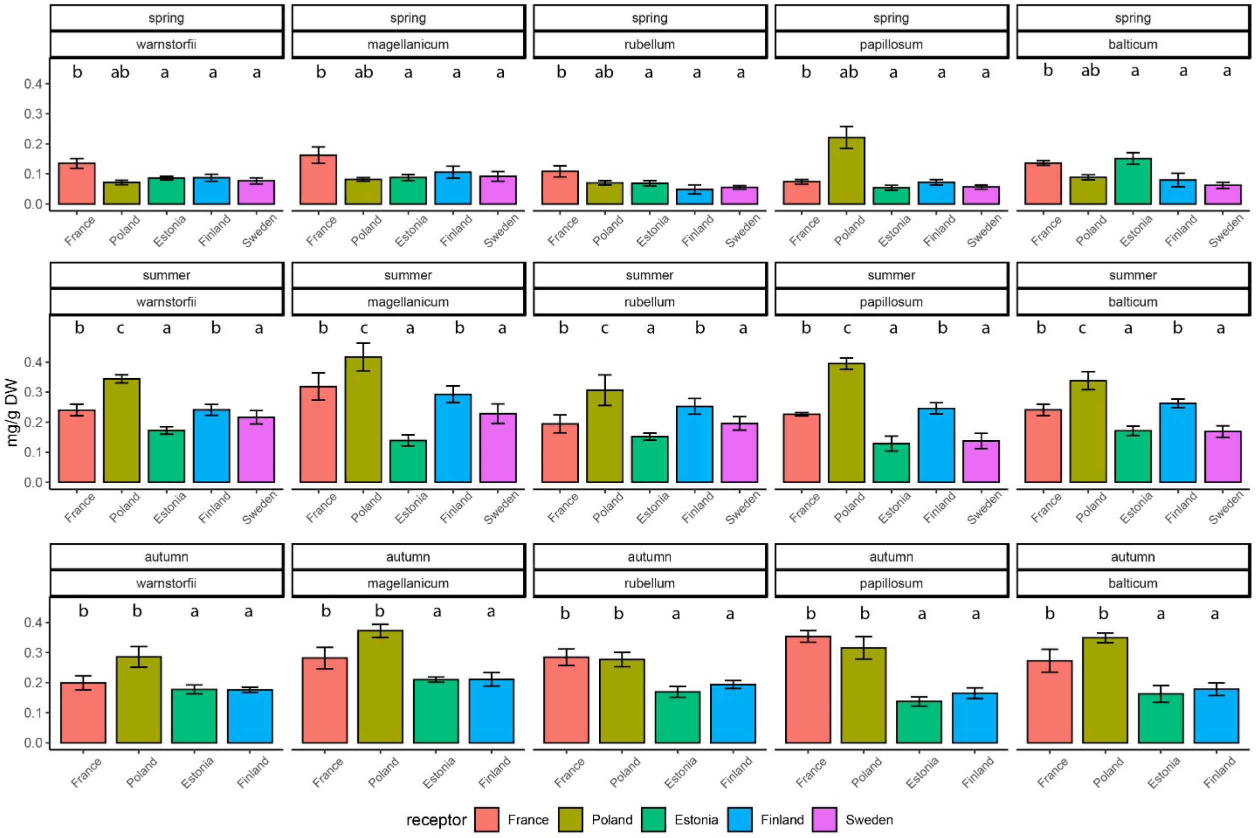

Figure S9. Chlorophyll *a* content in *Sphagnum* tissues collected in the transplanted mesocosms across seasons and sites. Data are mean ± standard errors (SE) (n = 5 independent plots per species and per site). Letters indicate significant differences at $P < 0.05$ (LME, post hoc test). warnstorfii: *Sphagnum warnstorfii*, magellanicum: *S. magellanicum*, rubellum: *S. rubellum*, papillosum: *S. papillosum*, balticum: *S. balticum*.

Fig. S10

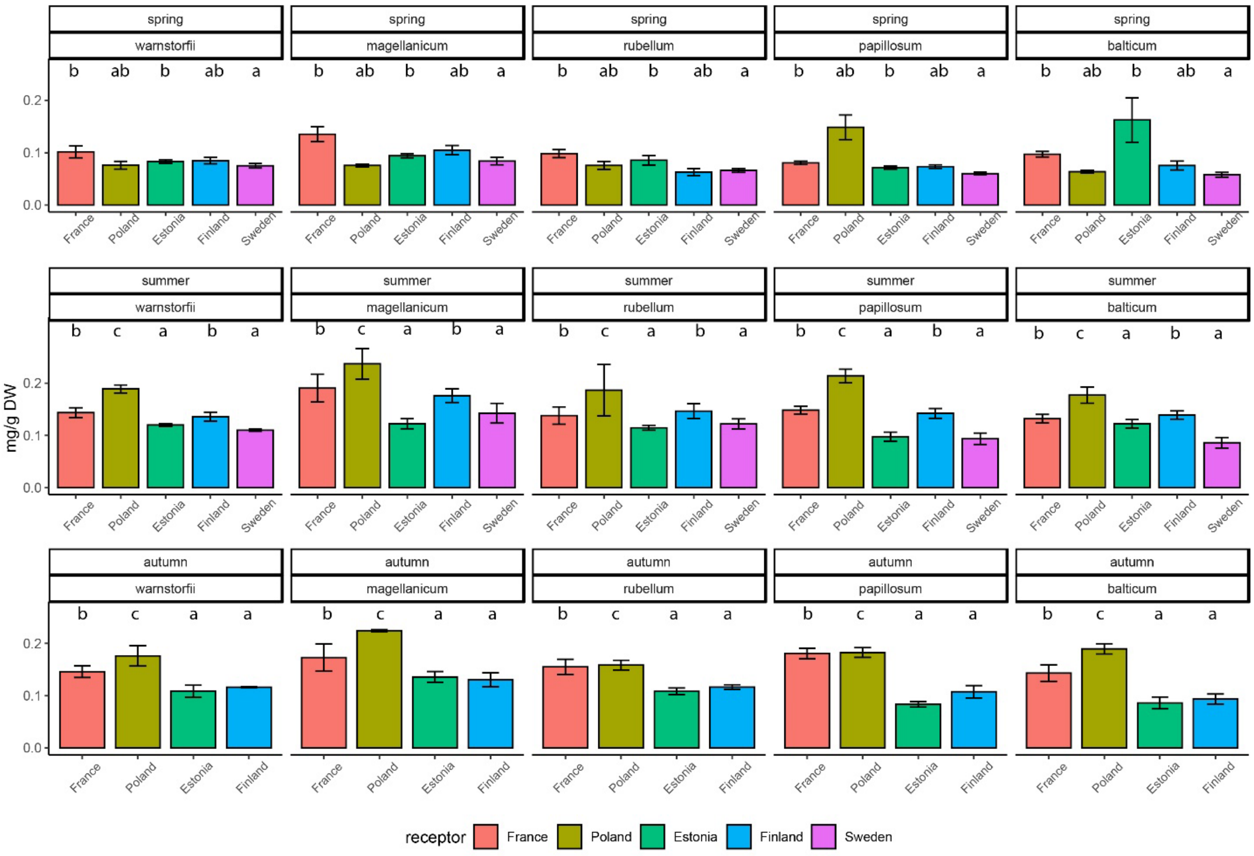

Figure S10. Chlorophyll *b* content in *Sphagnum* tissues collected in the transplanted mesocosms across seasons and sites. Data are mean ± standard errors (SE) (n = 5 independent plots per species and per site). Letters indicate significant differences at $P < 0.05$ (LME, post hoc test). warnstorfii: *Sphagnum warnstorfii*, magellanicum: *S. magellanicum*, rubellum: *S. rubellum*, papillosum: *S. papillosum*, balticum: *S. balticum*.

Fig. S11

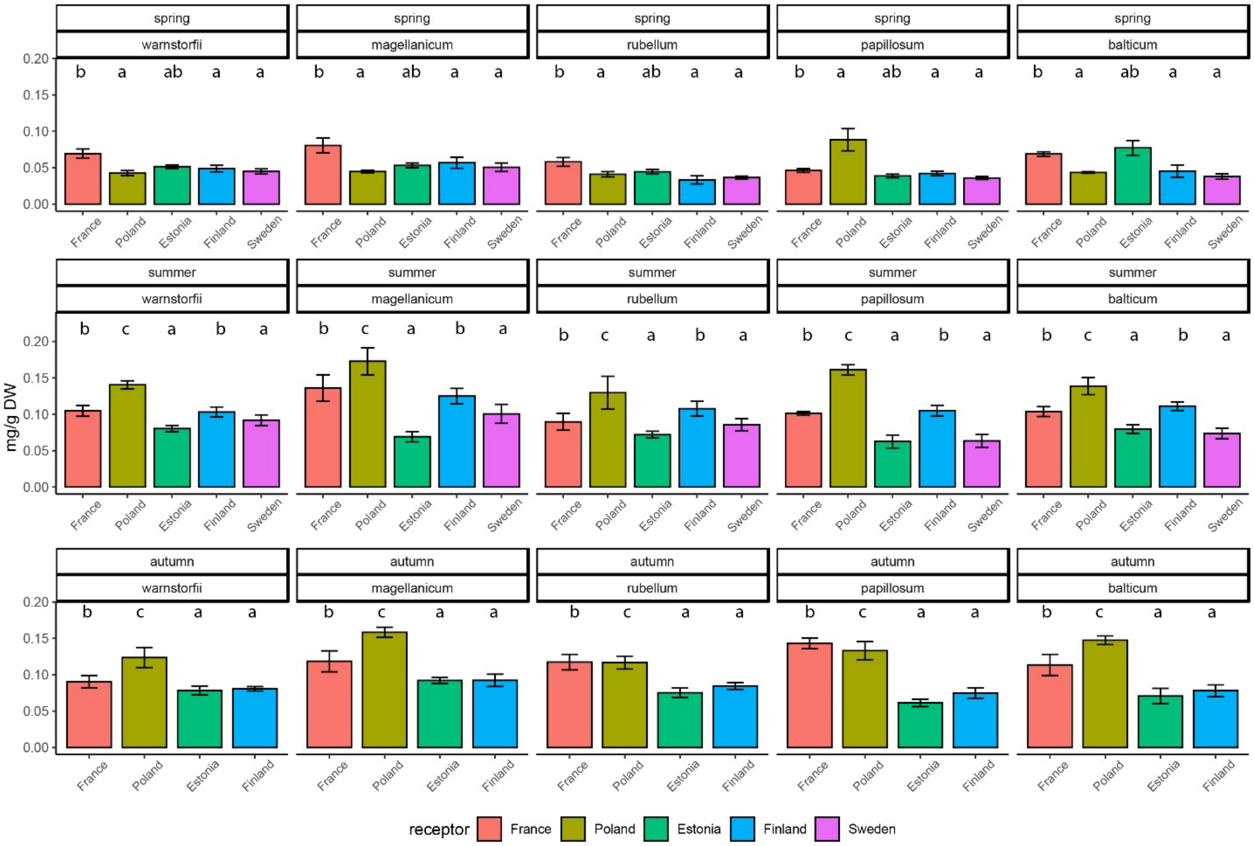

Figure S11. Carotenoid content in *Sphagnum* tissues collected in the transplanted mesocosms across seasons and sites. Data are mean ± standard errors (SE) (n = 5 independent plots per species and per site). Letters indicate significant differences at $P < 0.05$ (LME, post hoc test). warnstorfii: *Sphagnum warnstorfii*, magellanicum: *S. magellanicum*, rubellum: *S. rubellum*, papillosum: *S. papillosum*, balticum: *S. balticum*.

Fig. S12

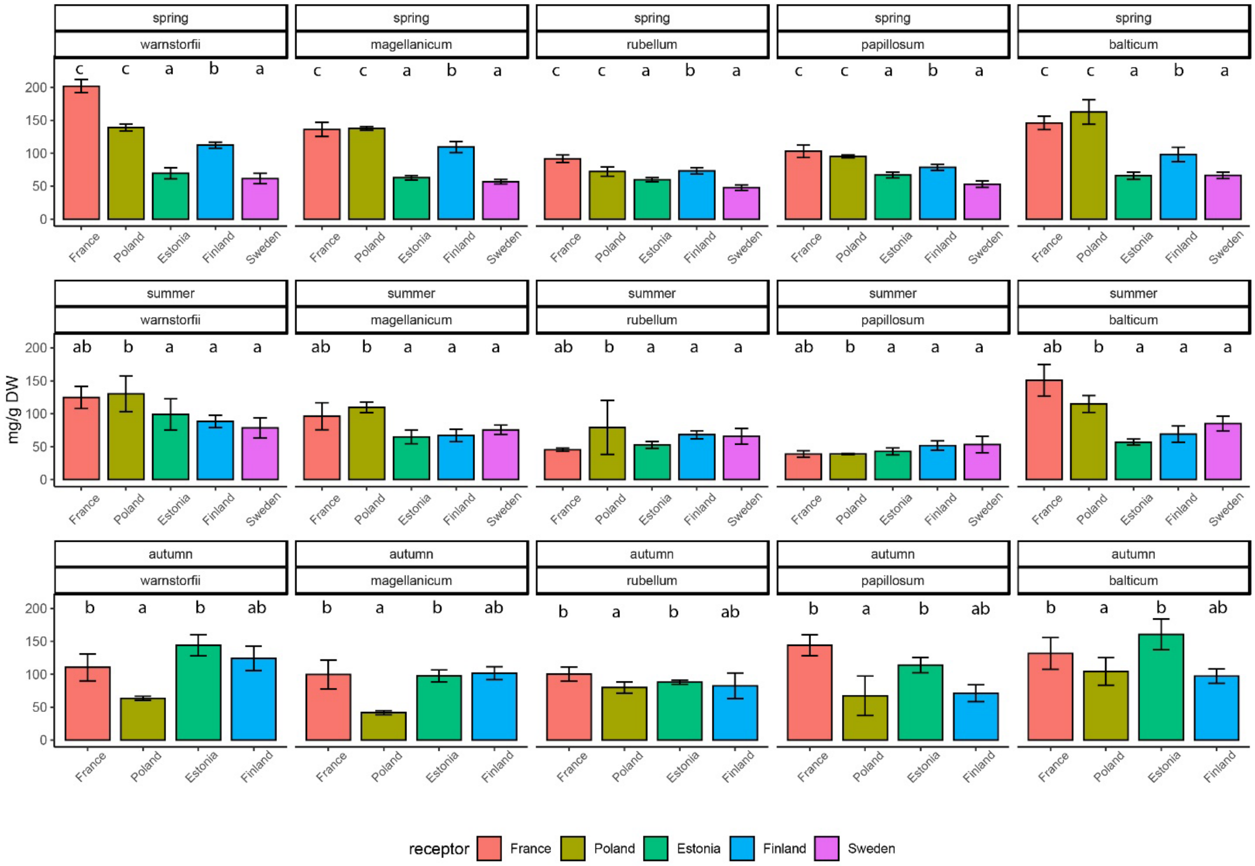

Figure S12. Carbohydrates content in *Sphagnum* tissues collected in the transplanted mesocosms across seasons and sites (n = 5). Data are mean ± standard errors (SE) (n = 5 independent plots per species and per site). Letters indicate significant differences at $P < 0.05$ (LME, post hoc test). warnstorfii: *Sphagnum warnstorfii*, magellanicum: *S. magellanicum*, rubellum: *S. rubellum*, papillosum: *S. papillosum*, balticum: *S. balticum*.

Fig. S13

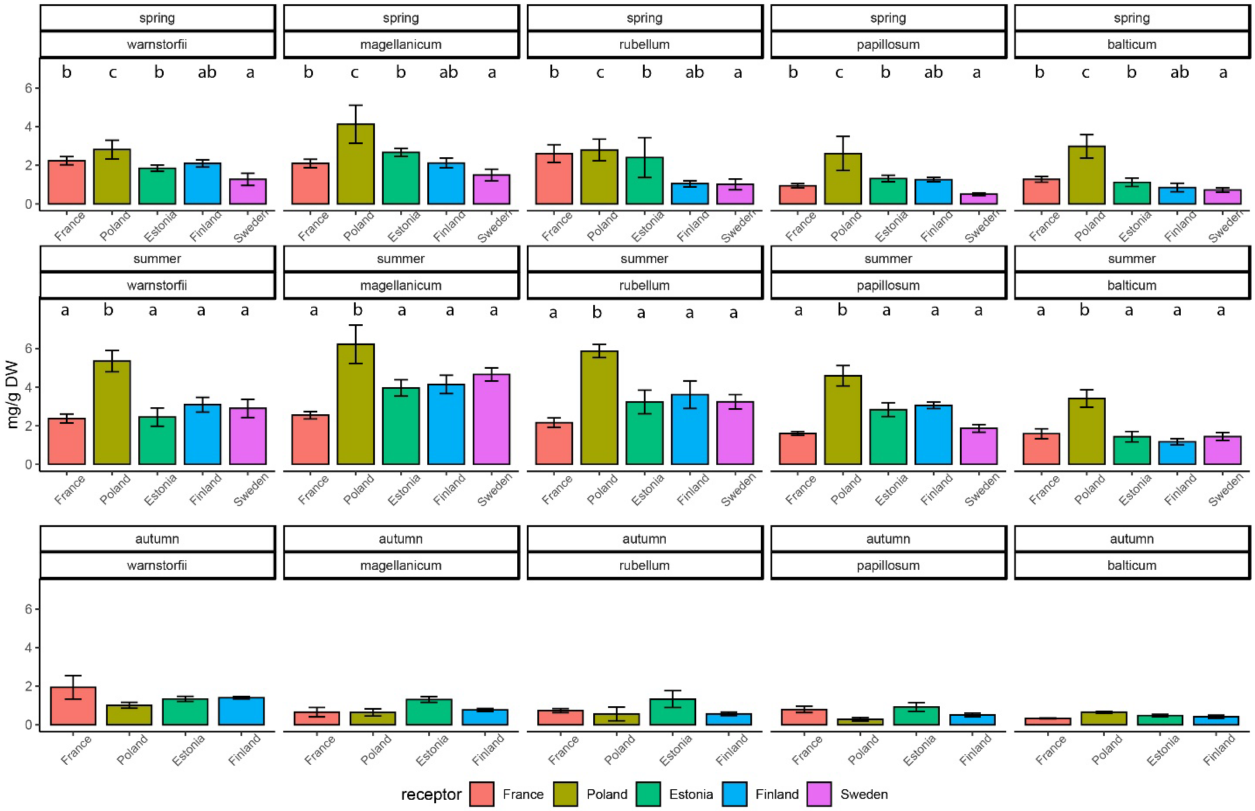

Figure S13. Water-soluble phenolic content in *Sphagnum* tissues collected in the transplanted mesocosms across seasons and sites (n = 5 independent plots per species and per site). Data are mean ± standard errors (SE). Letters indicate significant differences at $P < 0.05$ (LME, post hoc test). warnstorfii: *Sphagnum warnstorfii*, magellanicum: *S. magellanicum*, rubellum: *S. rubellum*, papillosum: *S. papillosum*, balticum: *S. balticum*.

Fig. S14

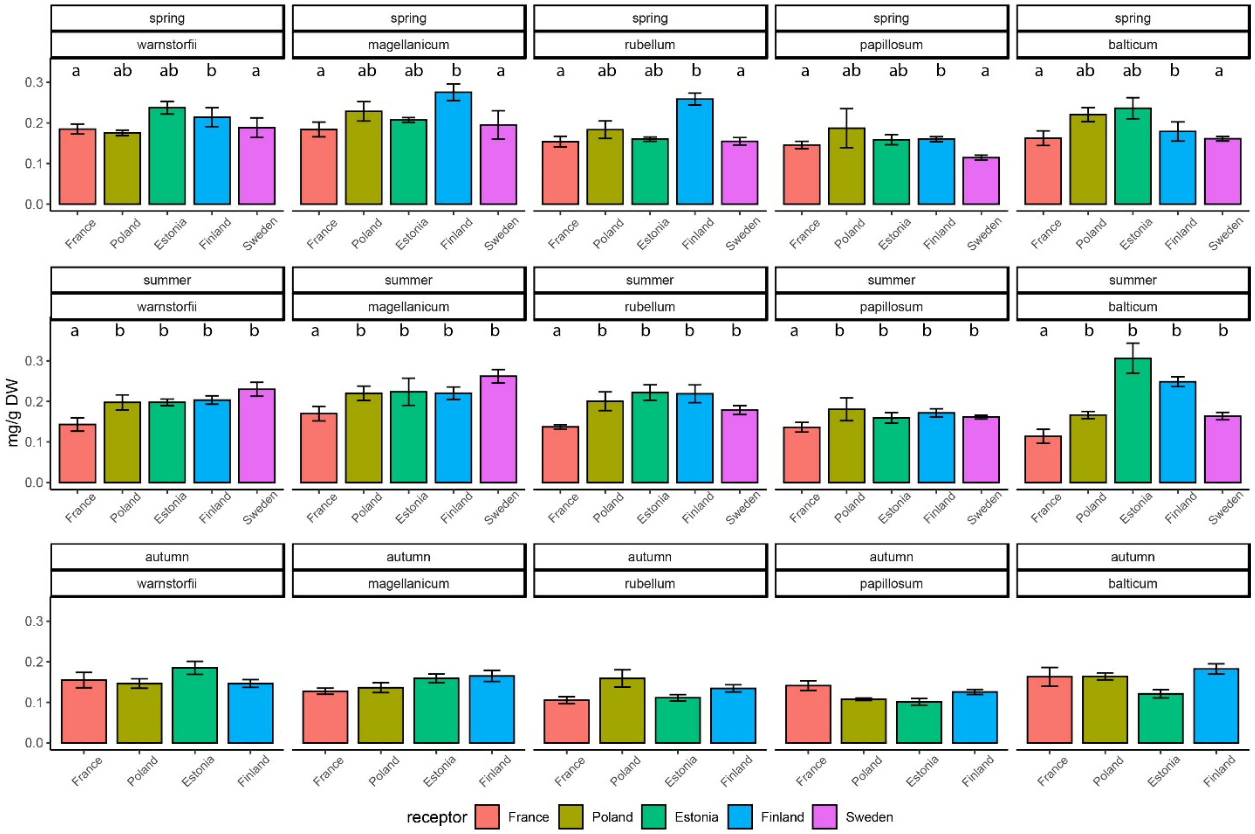

Figure S14. Proline content in *Sphagnum* tissues collected in the transplanted mesocosms across seasons and sites (n = 5 independent plots per species and per site). Data are mean ± standard errors (SE). Letters indicate significant differences at $P < 0.05$ (LME, post hoc test). warnstorfii: *Sphagnum warnstorfii*, magellanicum: *S. magellanicum*, rubellum: *S. rubellum*, papillosum: *S. papillosum*, balticum: *S. balticum*.

Fig. S15

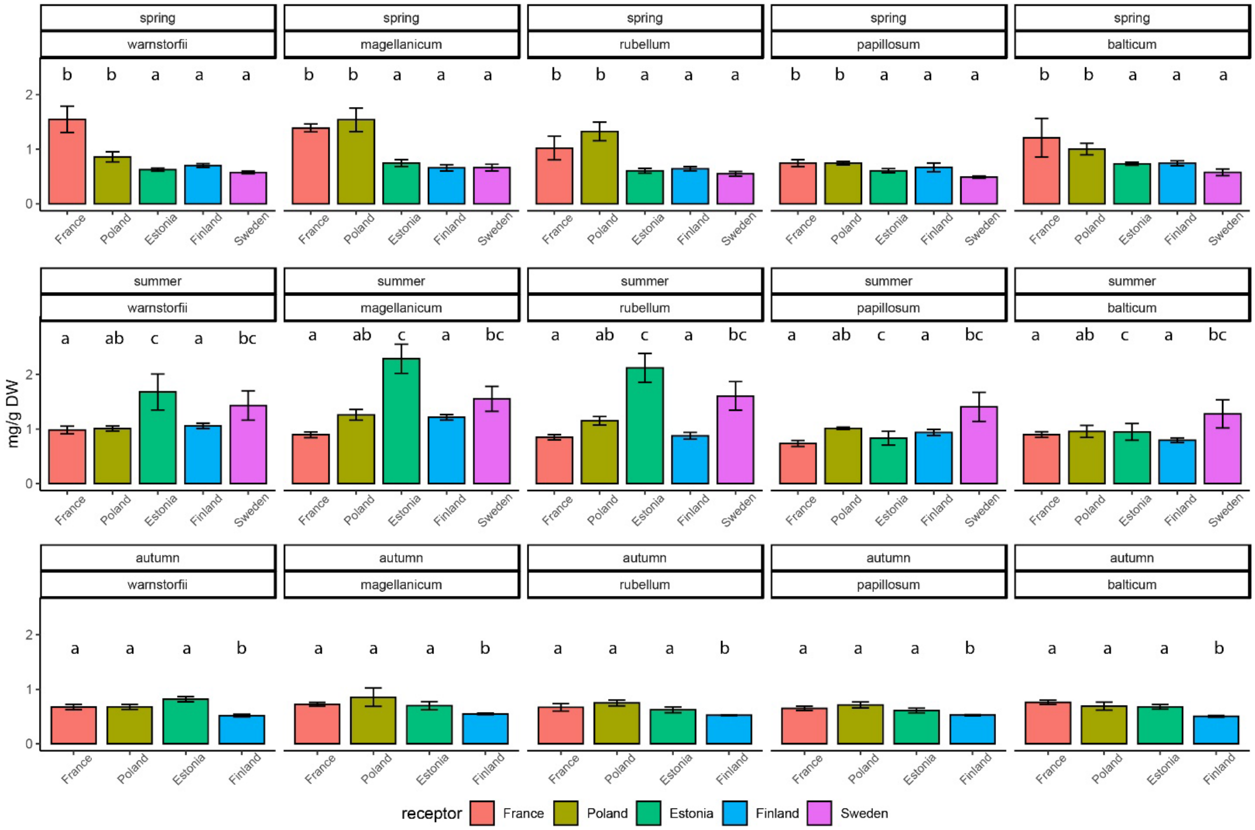

Figure S15. Tannin content in *Sphagnum* tissues collected in the transplanted mesocosms across seasons and sites (n = 5 independent plots per species and per site). Data are mean ± standard errors (SE). Letters indicate significant differences at $P < 0.05$ (LME, post hoc test). warnstorfii: *Sphagnum warnstorfii*, magellanicum: *S. magellanicum*, rubellum: *S. rubellum*, papillosum: *S. papillosum*, balticum: *S. balticum*.

Fig. S16

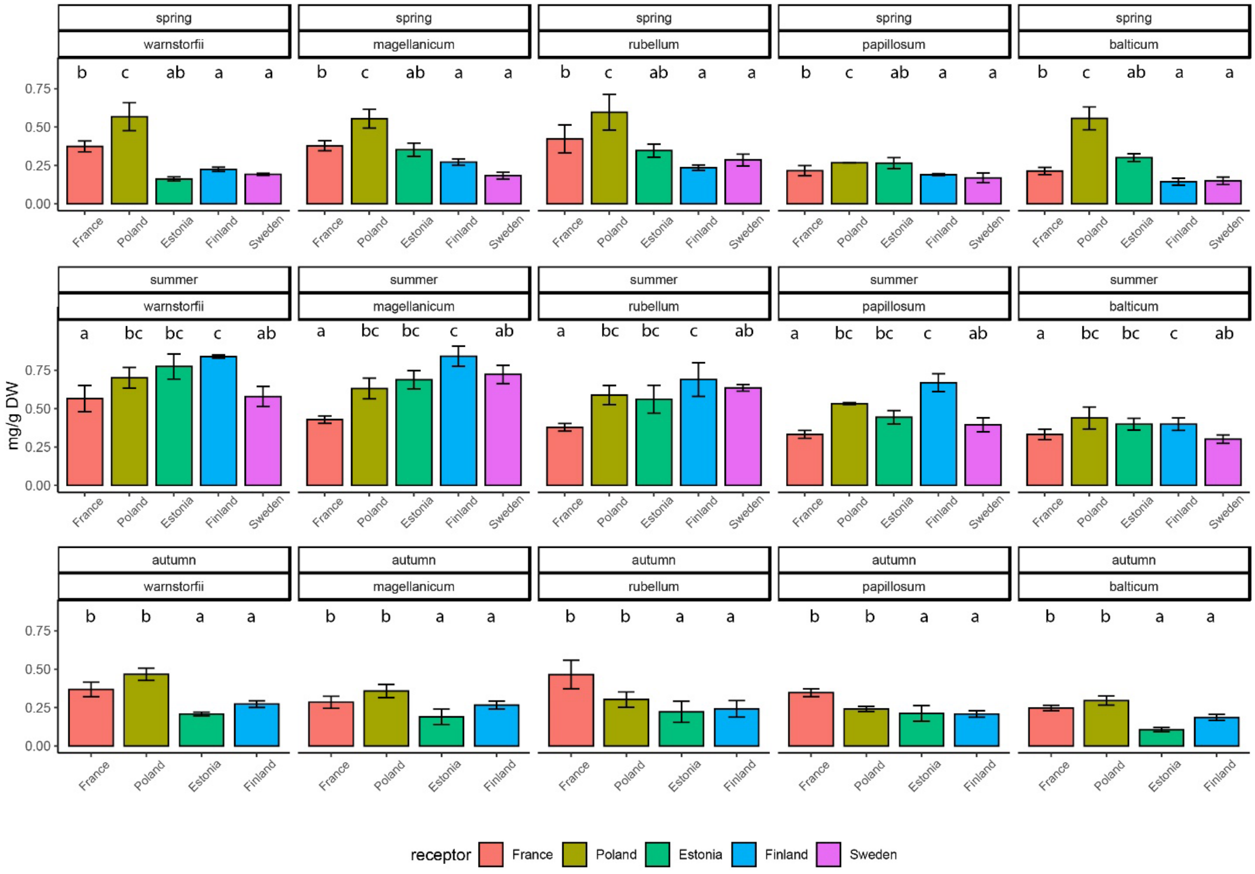

Figure S16. Total phenolic content in *Sphagnum* tissues collected in the transplanted mesocosms across seasons and sites (n = 5 independent plots per species and per site). Data are mean ± standard errors (SE). Letters indicate significant differences at $P < 0.05$ (LME, post hoc test). warnstorfii: *Sphagnum warnstorfii*, magellanicum: *S. magellanicum*, rubellum: *S. rubellum*, papillosum: *S. papillosum*, balticum: *S. balticum*.

Fig. S17

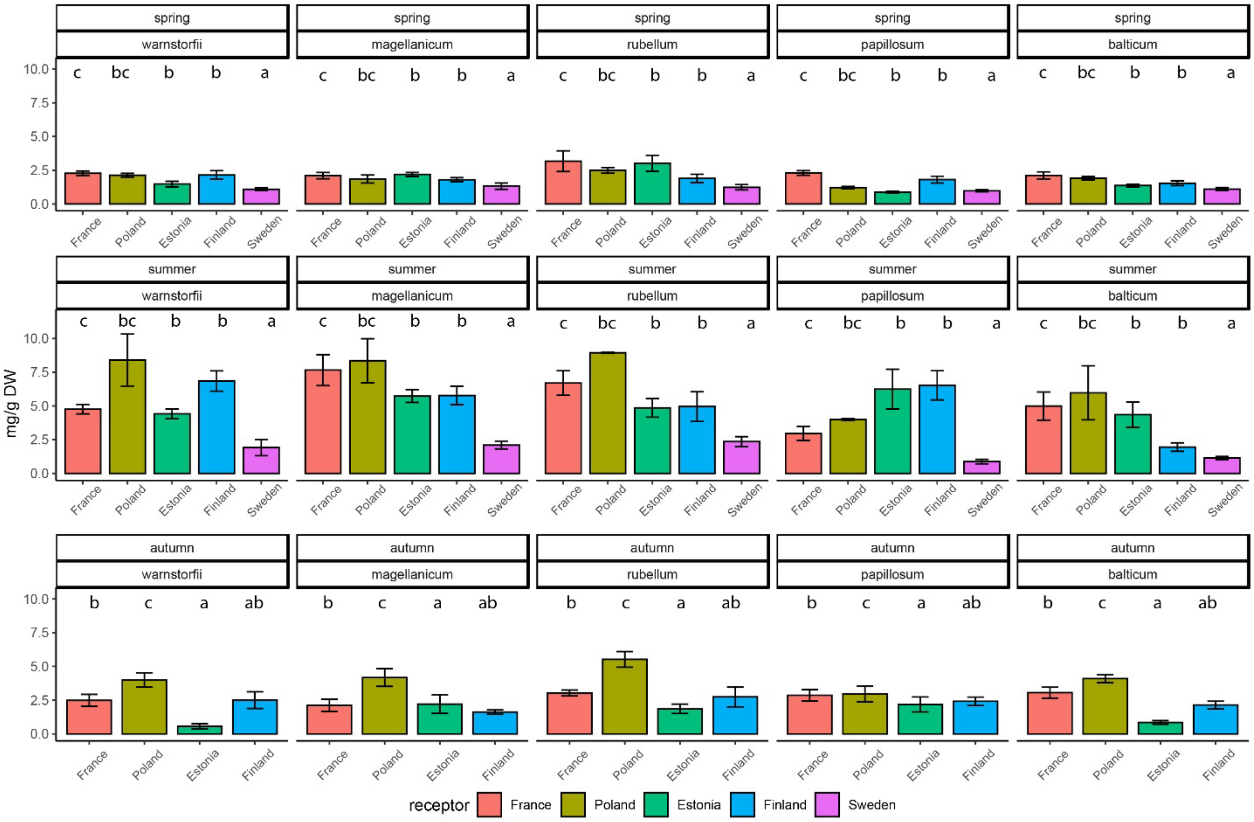

Figure S17. Flavonoid content in *Sphagnum* tissues collected in the transplanted mesocosms across seasons and sites (n = 5 independent plots per species and per site). Data are mean ± standard errors (SE). Letters indicate significant differences at $P < 0.05$ (LME, post hoc test). warnstorfii: *Sphagnum warnstorfii*, magellanicum: *S. magellanicum*, rubellum: *S. rubellum*, papillosum: *S. papillosum*, balticum: *S. balticum*.

Fig. S18

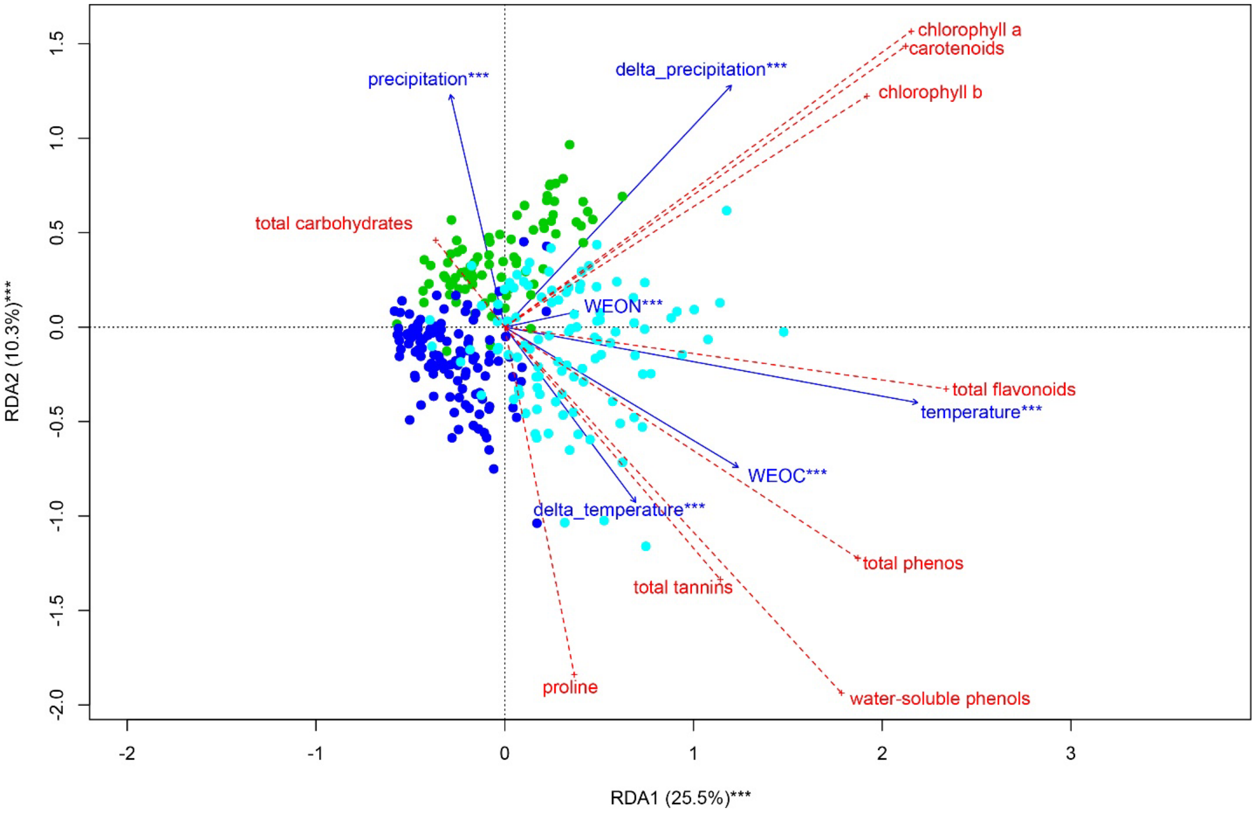

Figure S18. Redundancy Analysis (RDA) of metabolites in transplanted *Sphagnum* species over three seasons in relation to local (WEOC and WEON) and regional (temperature, precipitation) environmental variables. The transplant effect is tested using Δtemperature and Δprecipitation variables. Adjusted R^2= 0.35. Colours indicate the season: deep blue = spring, light blue = summer, green = autumn. Asterisks indicate significance of the axes and explanatory variables at *P < 0.05, ** P< 0.01, *** P < 0.001 following 1,000 permutations (ANOVAs). n = 125 independent plots per season.

Fig. S19

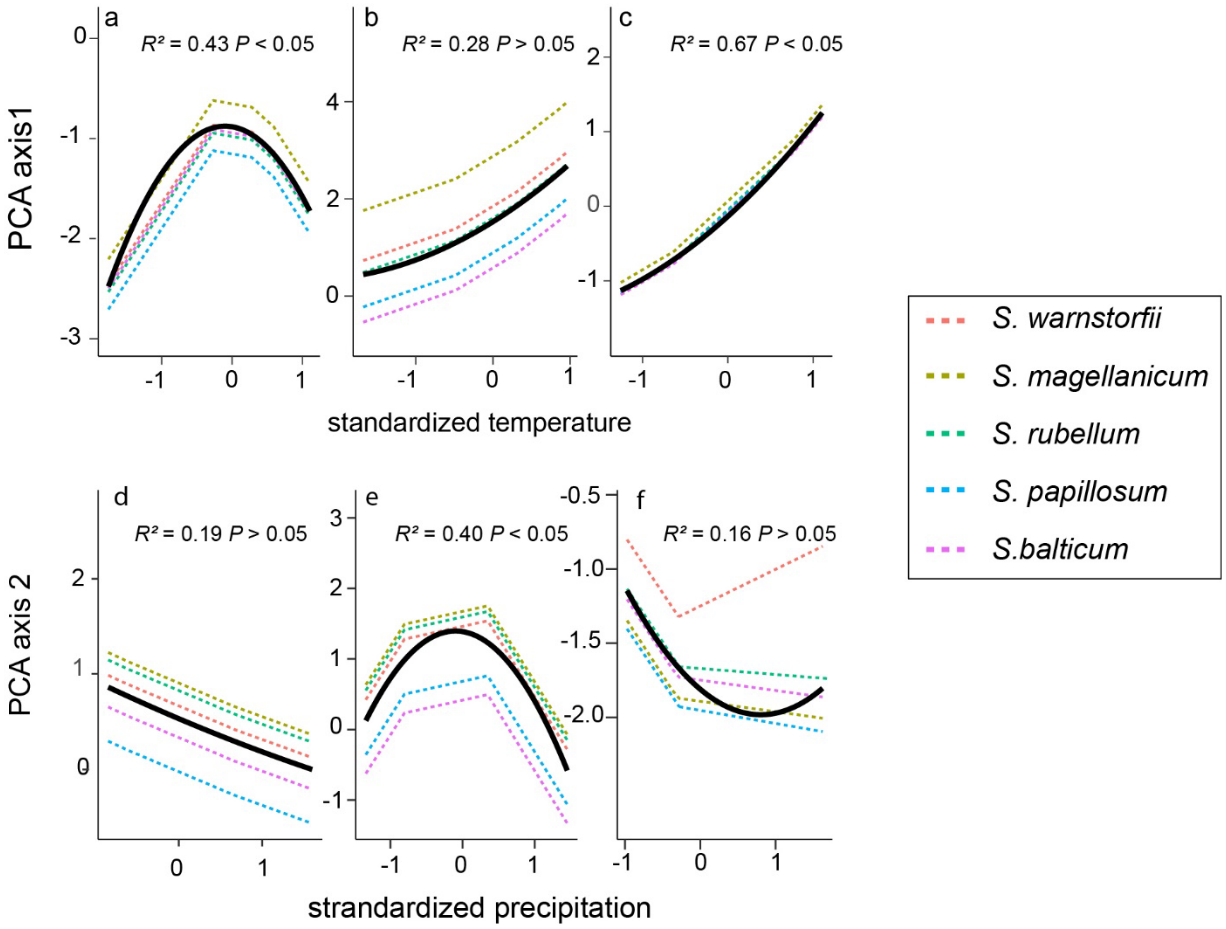

Fig. S19. Metabolic plasticity across mean-centred cumulative temperatures (a-c) and precipitation (d-f) of the receptor sites for the five *Sphagnum* species in spring (a, d), summer (b, e) and autumn (c, f). The thick black line represents the quadratic regression model fit of the overall effect of temperature or precipitation (the predicted average of five Sphagnum species) on *Sphagnum* metabolic composition and dashed coloured lines represent each *Sphagnum* species modelled reaction norms from the RRMM that accounts for differences in intercept (a-f) and slope (f).

Fig. S20

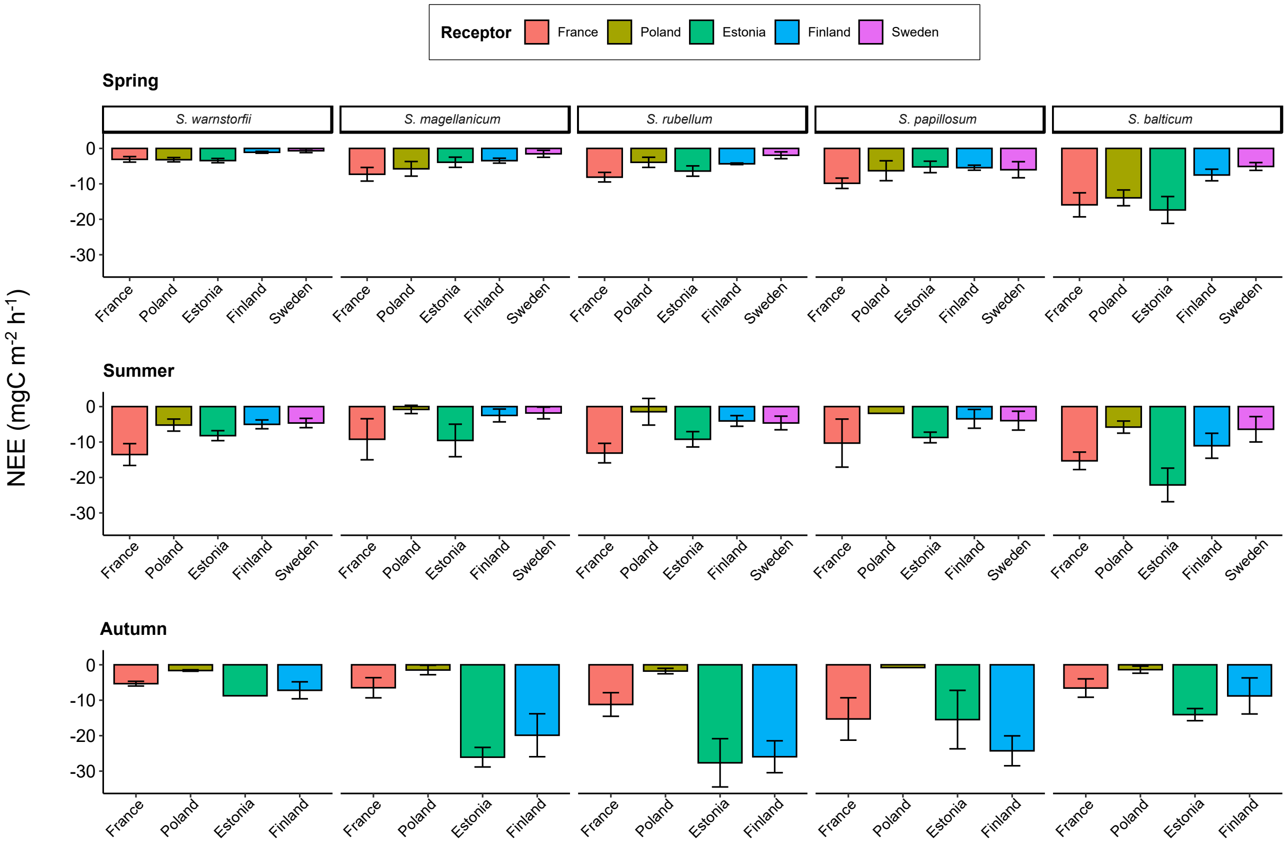

Figure S20: Net Ecosystem Exchange (NEE, mgC m^{-2} h^{-1}), standardized by vascular plant cover, in transplanted peat-mesocosms over three seasons. Receptor sites are coloured and dominant *Sphagnum* species are ordered according to the south-north gradient. Each value represents the mean ± SE (n = 5 independent plots per species and per site; n = 20 in autumn as peat-mesocosm in Sweden could not be sampled). Negative values show C uptake into the system, while positive values show C losses in the system.

Fig. S21

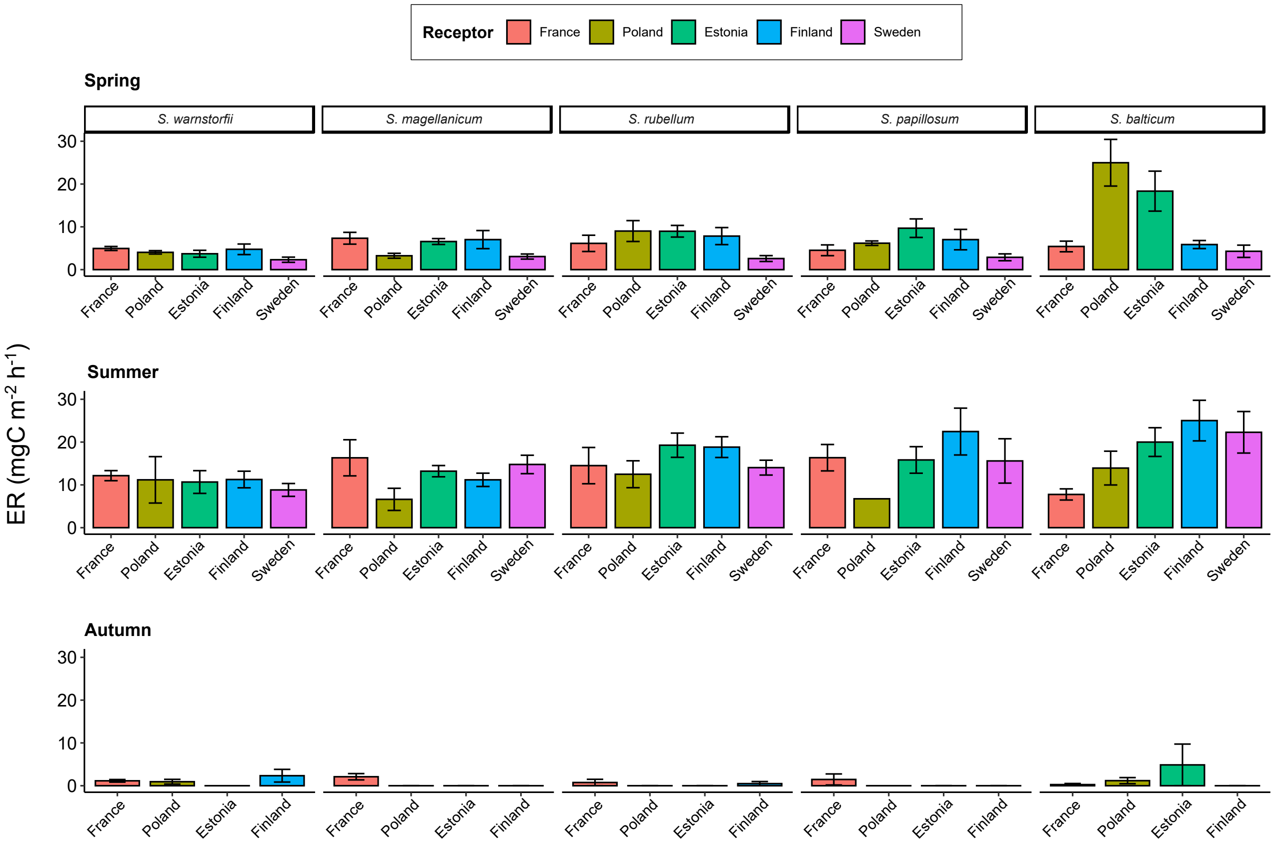

Figure S21: Ecosystem Respiration (ER, mgC m^{-2} h^{-1}), standardized by vascular plant cover, in transplanted peat-mesocosms over three seasons. Receptor sites are coloured and dominant *Sphagnum* species are ordered according to the south-north gradient. Each value represents the mean ± SE (n = 5 independent plots per species and per site; n = 20 in autumn as peat-mesocosm in Sweden could not be sampled).

Fig. S22

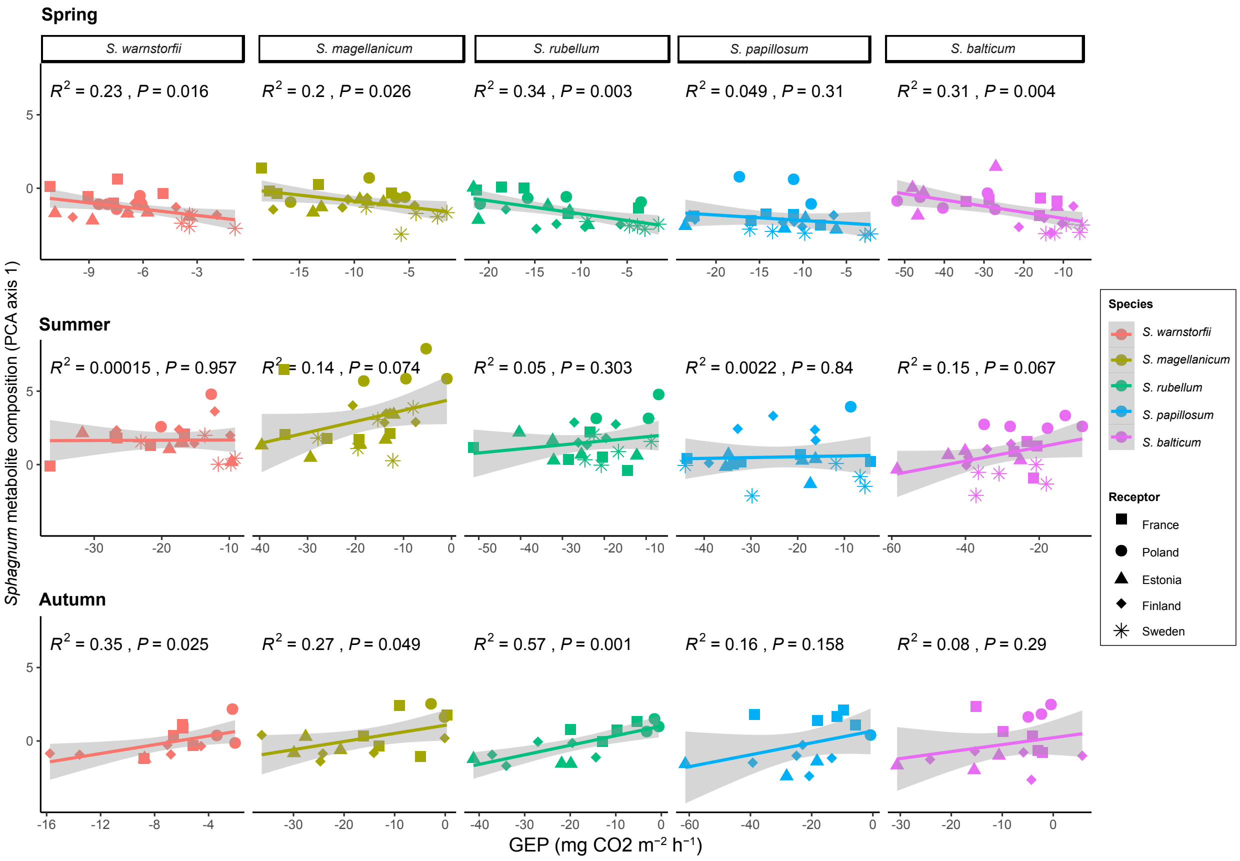

Figure S22. Correlation between the *Sphagnum* metabolite composition (PCA axis 1) and GEP (standardized values) in transplanted peat-mesocosms across seasons. Receptor sites are represented with different shapes and transplanted *Sphagnum* species with different colours. Both species and receptor sites are ordered according to the south-north gradient (from France to Sweden). n = 25 independent plots per species across sites (n = 20 independent plots in autumn as peat-mesocosm in Sweden could not be sampled).

Fig. S23

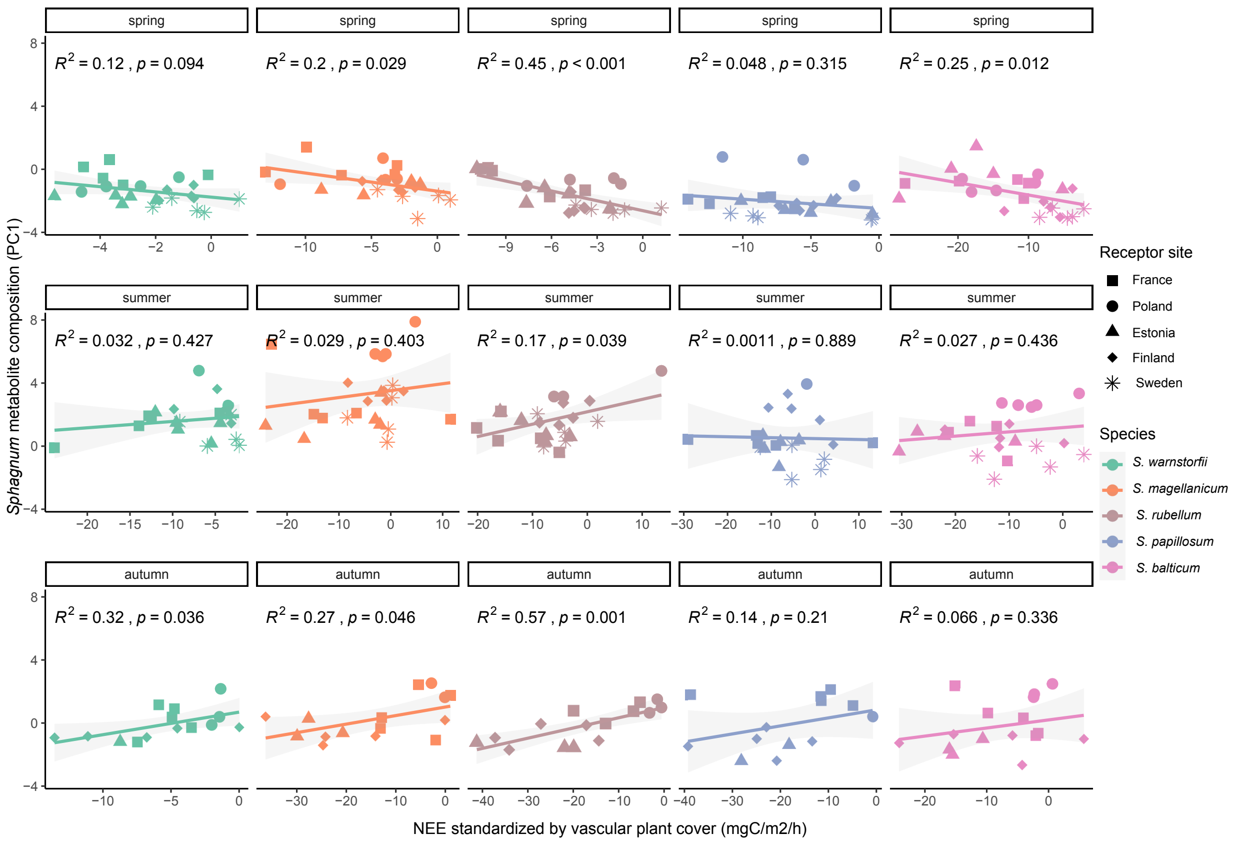

Figure S23. Correlation between the *Sphagnum* metabolite composition (PCA axis 1) and NEE (standardized values) in transplanted peat-mesocosms across seasons. Receptor sites are represented with different shapes and transplanted *Sphagnum* species with different colours. Both species and receptor sites are ordered according to the south-north gradient (from France to Sweden). n = 25 independent plots per species across sites (n = 20 independent plots in autumn as peat-mesocosm in Sweden could not be sampled).

Fig. S24

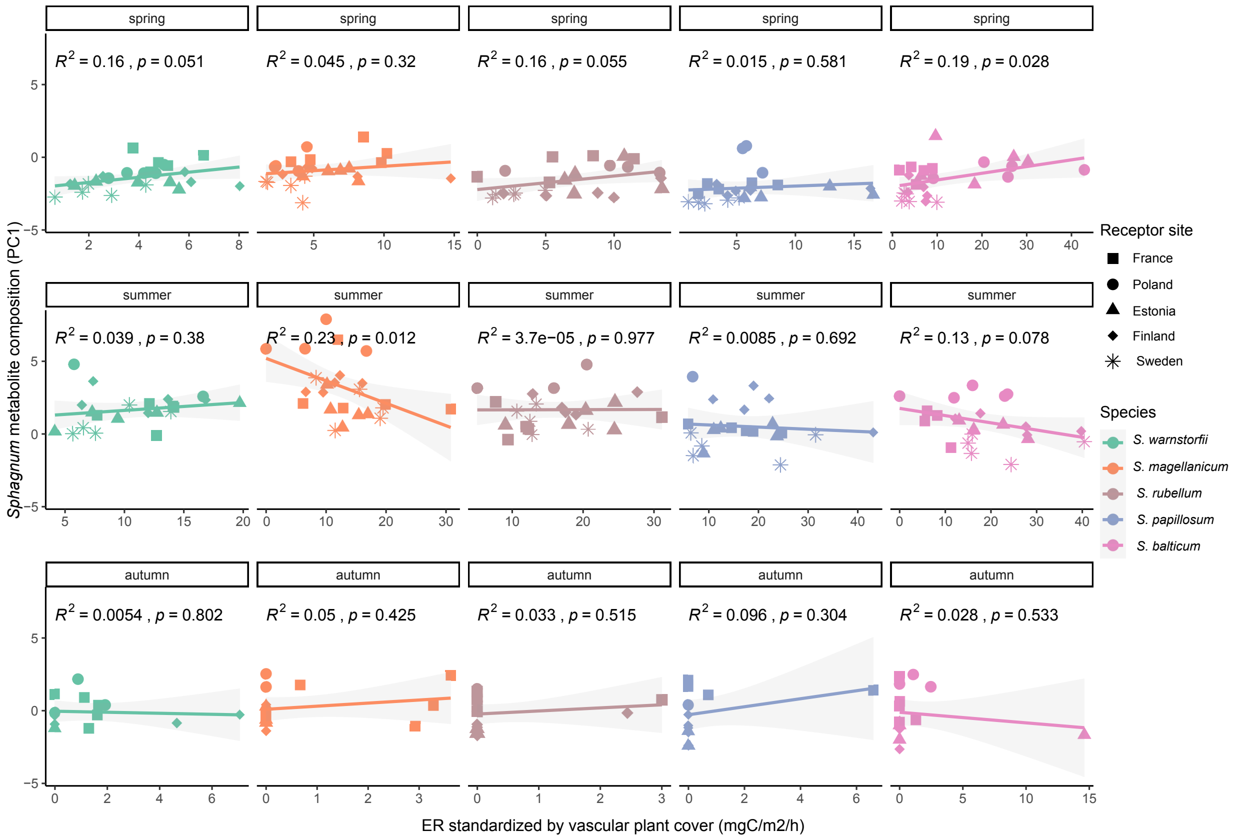

Figure S24. Correlation between the *Sphagnum* metabolite composition (PCA axis 1) and ER (standardized values) in transplanted peat-mesocosms across seasons. Receptor sites are represented with different shapes and transplanted *Sphagnum* species with different colours. Both species and receptor sites are ordered according to the south-north gradient (from France to Sweden). n = 25 independent plots per species across sites (n = 20 independent plots in autumn as peat-mesocosm in Sweden could not be sampled).
